# Behavior of Auramine O in the Aqueous Solution of Two Kolliphors and Their Mixture

**DOI:** 10.3390/molecules27238493

**Published:** 2022-12-02

**Authors:** Katarzyna Szymczyk, Andrzej Lewandowski, Anna Zdziennicka, Magdalena Szaniawska, Bronisław Jańczuk

**Affiliations:** Department of Interfacial Phenomena, Institute of Chemical Sciences, Faculty of Chemistry, Maria Curie-Skłodowska University in Lublin, Maria Curie-Skłodowska Sq. 3, 20-031 Lublin, Poland

**Keywords:** adsorption, micellization, solubilization, surface tension, contact angle

## Abstract

The studies on the behavior of Auramine O (AuO) at the water–air interface and in the bulk phase of the aqueous solution of Kolliphor^®^ ELP (ELP) and Kolliphor^®^ RH 40 (RH40) and their mixture were based on the results obtained from the measurements of the contact angle of water, formamide and diiodomethane on the polytetrafluoroethylene covered by the AuO layer, the surface tension of the aqueous solution of AuO, AuO + ELP, AuO + RH40, AuO + ELP + RH40, density and fluorescence intensity. Based on the obtained results, it was possible to determine components and parameters of the AuO surface tension, concentration and composition of the mixed monolayer, including AuO, ELP and RH40, as well as that of the mixed micelles, and to determine the Gibbs standard free energy of adsorption, micellization and AuO solubilization. The obtained results also showed that surface tension isotherms of the studied solutions can be described by the Szyszkowski equation and the exponential function of the second order and predicted by the Fainerman and Miller equation. In addition, the mixed surface layer composition can be predicted based on the contribution of the components of this layer to the water surface tension reduction.

## 1. Introduction

Auramine O (4,4′-(Imidocarbonyl)bis(*N*,*N*-dimethyaniline) monochydrochloride) (AuO) is an organic compound that is a cationic dye, and its salts are often used in the leather, textile and paper industries [1,2,3]. AuO also can be applied as a fluorescent probe [4,5]. Unfortunately, due to its toxicity AuO is dangerous to the environment [6]. Therefore, it is important to treat industrial wastewater containing AuO [7,8]. The wastewater treatment often proceeds by adsorbing AuO on the surface of the activated carbon [1,9]. Practical application of AuO and its removal from the natural environment are not based on sufficient knowledge of its physicochemical properties such as the tendency of AuO to adsorb at different interfaces, adhesion to the solid surfaces and solubilization in the surfactant micelles. The process of the AuO solubilization in the surfactant micelles can be of particular importance if it is used as a fluorescent probe [4,5]. It is essential to know the extent of the AuO effect on the adsorption and aggregation of the surfactants and their mixtures. This seems to be especially important in the case of high molecular weight surfactants that can interact extensively with the AuO molecules.

Kolliphor^®^ ELP (ELP) and Kolliphor^®^ RH 40 (RH40), whose main constituent is triricinoleate ester of ethoxylated glycerol, can be considered as such a kind of the surfactants. The large hydrophobic tail of the ELP and RH40 molecules includes three hydrocarbon chains with one –OH group in each chain. The hydrophilic head of these surfactants includes oxyethylene groups [10,11]. Due to the large contact area of the hydrophobic parts of ELP and RH40 molecules, it is possible that strong interactions can occur between these parts and the AuO molecules, resulting in the great effect of AuO on the adsorption and aggregation of ELP and RH40 as well as their mixtures. This conclusion was related to the purpose of our studies, which were based on the contact angle measurements of the water, formamide and diiodomethane on polytetrafluoroethylene (PTFE) covered by an AuO layer, the measurements of the surface tension and density of the aqueous solutions of AuO, the mixture of AuO with ELP or RH40, as well as the mixture of AuO with ELP and RH40 at a mole fraction of ELP in the bulk phase equal to 0.8 at T = 293 K. For the mixture of AuO and ELP, the fluorescence intensity was also measured at temperatures from 293 to 318 K. The obtained values of the contact angle were used for the components and parameters of the AuO surface tension determination, taking into account the van Oss et al. [12,13,14] approach to the surface and interface tensions. The results of the surface tension measurements were considered with regard to the adsorption of AuO and its mixtures with ELP and/or RH40, the composition of the mixed monolayer at the water–air interface and the mixed aggregates formation, as well as standard Gibbs free energy of the adsorption and micellization. The composition of the mixed monolayer at the water–air interface and mixed micelles was analyzed using the modified Hua and Rosen equation [15,16] and also was based on the contribution of a particular component of the mixture to the water surface tension reduction. This contribution was also taken into account in predicting the surface tension isotherms of the aqueous solution of the studied mixtures based on the isotherms of the solution of particular mixture components. The surface tension isotherms of the solutions of the studied mixtures were also analyzed using the Szyszkowski equation [15]. The density of the solution was considered with regard to the size of the particular components of the mixture. However, based on the fluorescence spectra, the solubilization of AuO in the ELP was taken into account.

## 2. Results and Discussion

### 2.1. Surface and Volumetric Properties of AuO, ELP and RH40

Knowledge of the surface and volumetric properties of AuO, ELP and RH40 can help understand the AuO behavior in the aqueous solution of surfactants and their mixtures. The properties include, among others, the surface tension, molecules volume, contact area of these molecules and ability to adsorb at different interfaces and to aggregate in the aqueous media.

**Figure 1:**
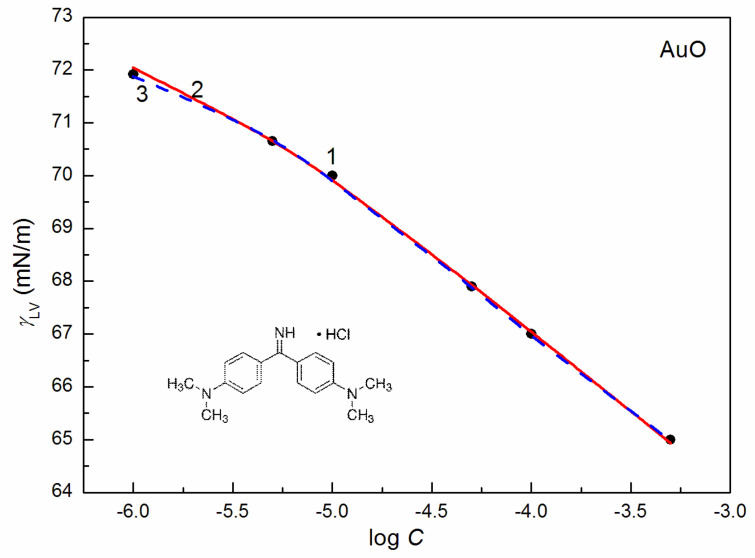
A plot of the surface tension (*γ_LV_*) of aqueous solutions of AuO vs. the logarithm of its concentration (log *C*). Point 1 corresponds to the measured values, curves 2 and 3 correspond to the values calculated from Equations (5) and (6), respectively.

The surface tension of a given compound can be determined, among others, from the contact angle values of the model liquids in the properly chosen systems [12,13,14]. However, the determination of the surface tension and its components and parameters for the compounds having big molecules, such as the surfactants, is more complicated. According to van Oss and Constanzo [14], the surface tension of such compounds depends on the way of their molecules’ orientation toward the air phase. Hence, in the case of surfactants the concept of surface tension of the tail and head appeared. The application of this concept to AuO is difficult. The AuO molecule is composed of four –CH_3_ groups, two benzene rings and one amino group (Figure 1). The –CH_3_ and benzene units are hydrophobic. However, they can interact with the adherent medium by the weak hydrogen bonds’ formation due to the free electron presence. In fact, there is only one distinct hydrophilic group in the AuO molecule, namely –NH_2_. This fact and the distribution of individual chemical groups in the AuO molecule suggest that its surface tension should be a result of mainly the Lifshitz–van der Waals intermolecular interactions. However, it is difficult to find tail and head in the AuO molecule. Mentioned suggestions were confirmed by the calculation of the components and parameters of the AuO surface tension using the contact angle of model liquids measured on the PTFE surface covered by the AuO layer in the van Oss et al. equation having the form [12,13,14]:

(1) $$\gamma_{LV}\left(cos\theta +1\right)=2\left(\sqrt{\gamma_{SV}^{LW}\gamma_{LV}^{LW}}+\sqrt{\gamma_{SV}^{+}\gamma_{LV}^{-}}+\sqrt{\gamma_{SV}^{-}\gamma_{LV}^{+}}\right),$$

where *θ* is the contact angle, *γ* is the surface tension, *LV* and *SV* indices refer to the liquid and solid, respectively, and the indices *LW*, + and − refer to the Lifshitz–van der Waals component and electron-acceptor and electron-donor parameters of the surface tension of the liquid and the solid, respectively.

For calculation of the components and parameters of the AuO surface tension, the contact angle of water (60°), formamide (47°) and diiodomethane (40°) measured on the PTFE surface covered by AuO layer as well as the component and parameters of these liquids’ surface tension taken from the literature were used [17]. The obtained values of $\gamma_{SV}^{LW}$, $\gamma_{SV}^{+}$ and $\gamma_{SV}^{-}$ are equal to 39.61, 0.0066 and 20.30 mN/m, respectively. The total AuO surface tension was calculated from the following equation [12,13,14]:

(2) $$\gamma_{SV}=\gamma_{SV}^{LW}+\gamma_{SV}^{AB}=\gamma_{SV}^{LW}+2\sqrt{\gamma_{SV}^{+}\gamma_{SV}^{-}},$$

where $\gamma_{SV}^{AB}$ is the acid–base component of the solid surface tension.

The surface tension of AuO calculated from Equation (2) is equal to 40.34 mN/m. This value is close to that of polymethyl methacrylate (PMMA) and to the other compounds with the benzene units in the molecules [17]. As follows from the presence of different chemical groups in the AuO molecule, the surface tension of AuO practically originates from the LW intermolecular interactions. In turn, in contrast to AuO the molecules of ELP and RH40 have the strong hydrophilic and hydrophobic parts. The determination of the surface tension components and parameters of ELP and RH40 is not easy. It was found that the minimal surface tension of the aqueous solution of surfactants satisfies the condition [10]:

(3) $$\gamma_{LV}=\gamma_{S}X_{S}+\gamma_{W}X_{W},$$

where *X_S_* and *X_W_* are the fraction of the interface area occupied by the surfactant and water, and *γ_S_* and *γ_W_* are the surface tension of the surfactant tail and water.

The value of *X_S_* can be expressed by *Γ^max^*/*Γ*^∞^. Hence expression (Equation (3)) assumes the form:

(4) $$\gamma_{LV}=\gamma_{S}\;\frac{\Gamma^{max}}{\Gamma^{\infty }}+\gamma_{W}\left(1-\frac{\Gamma^{max}}{\Gamma^{\infty }}\right),$$

where *Γ^max^* and *Γ*^∞^ are the maximal and limiting concentrations in the monolayer at the water–air interface, respectively.

Taking into account the values of *γ_W_*, *γ_LV_*, *Γ^max^* and *Γ*^∞^ from the literature [10], the *γ_S_* values were calculated. These values for ELP and RH40 are equal to 36.0 and 35.2 mN/m, respectively. These values for ELP and RH40 are almost the same. This is consistent with the fact that both surfactant molecules have the same tail. However, the *γ_S_* values for both surfactants are higher than that of the LW component of the glycerol surface tension [17]. The hydrophilic part of the ELP and RH40 molecules is composed of oxyethylene groups and their number is different in the ELP and RH40 molecules. It seems that the surface tension of the head of these surfactants can be close to that of Triton X-165 (TX165). This conclusion is in agreement with the Fowkes theory [18]. This theory shows that the surface tension of the compounds having big molecules does not depend on the interactions between molecules but on the interactions between the chemical groups being in the molecule and the distance between them. As a matter of fact, not only the components and parameters of the compound surface tension determine their adsorption at different interfaces and micellization but also their size and contactable area at the interface and between different molecules in the solution. The contactable area of the surfactants in the aqueous solution is closely associated with the number of water molecules being in the contact with the surfactant ones. For this reason, the volume and contactable area of AuO, ELP and RH40 were considered.

The earlier studies proved that the volume and contactable area of molecules can be calculated based on the length of the bonds between different atoms in the molecule, the angle between these bonds and the average distance between the molecules. The size of the molecule as well as some chemical groups of the molecule determined in this way can be described by means of the appropriate cube. The volume of this cube or the sum of cubes gives the volume of the molecule and contactable area [19]. The volume of the AuO molecule determined in such a way is equal to 479.31 Å^3^. This volume corresponds to the molar volume of AuO equal to 285.2 cm^3^/mol. The density calculated based on this value is equal to 1.065 g/cm^3^ being close to that reported in the literature (1.07 g/cm^3^) [20]. The contactable area of the AuO molecule at its parallel orientation at interface is equal to 141.16 Å^2^. The volume and the contactable area of ELP and RH40 were discussed earlier [10]. The comparison of the AuO molecule volume with those of ELP and RH40 molecules shows that 10 and 9 AuO molecules correspond to one molecule of RH40 and ELP, respectively. Moreover, the contactable area of the hydrophobic part of the ELP and RH40 molecules at their parallel orientation toward the interface is 2.4 times greater than that of the contactable area of AuO molecule. This fact can be important while considering of the behavior of AuO in the aqueous solution of ELP and RH40 as well as their mixture.

### 2.2. Surface Tension

The volume of the molecules of a given compound and its contactable area, surface tension as well as the kind of the intermolecular interactions from which this tension results decide about the adsorption of this compound at the water–air interface. This adsorption causes the change of the water surface tension. The surface tension of AuO results mainly from LW intermolecular interactions and therefore it is slightly soluble in water. It is possible that AuO forms the layer at the water–air interface in which its molecules are present only in the air phase. This phenomenon is probably similar to formation, for example, of the benzene layer on the water surface during its spreading [21,22,23]. The minimal surface tension of the aqueous solution of AuO that was possible to obtain is comparable to that of the water covered with the thin layer of benzene (Figure 1) [23]. The LW component of the AuO surface tension is greater than that of the water one (26.85 mN/m) [17]. Thus, it is impossible to reduce the LW component of the water surface tension due to the AuO layer. The changes of the water surface tension affected by this layer are related only to the decrease of the acid–base component (AB) of *γ_W_*.

**Figure 2:**
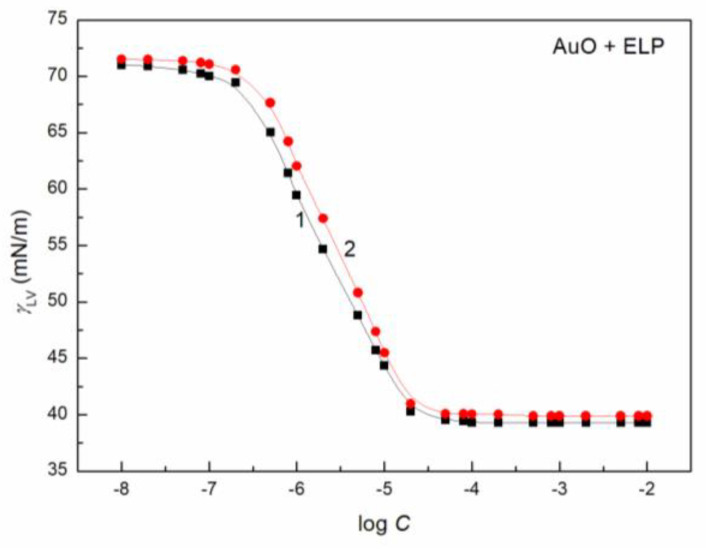
A plot of the surface tension (*γ_LV_*) of aqueous solutions of ELP (curve 1, squares) and AuO + ELP mixture (curve 2, circles) vs. the logarithm of ELP concentration (log *C*).

**Figure 3:**
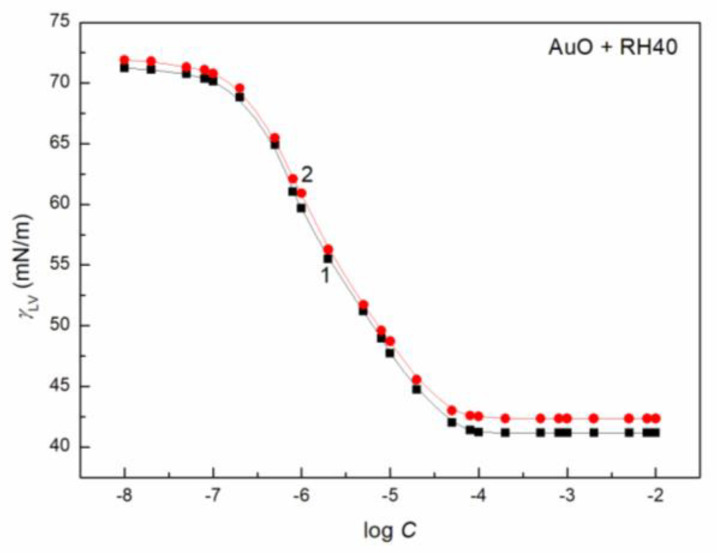
A plot of the surface tension (*γ_LV_*) of aqueous solutions of RH40 (curve 1, squares) and AuO + RH40 mixture (curve 2, circles) vs. the logarithm of RH40 concentration (log *C*).

**Figure 4:**
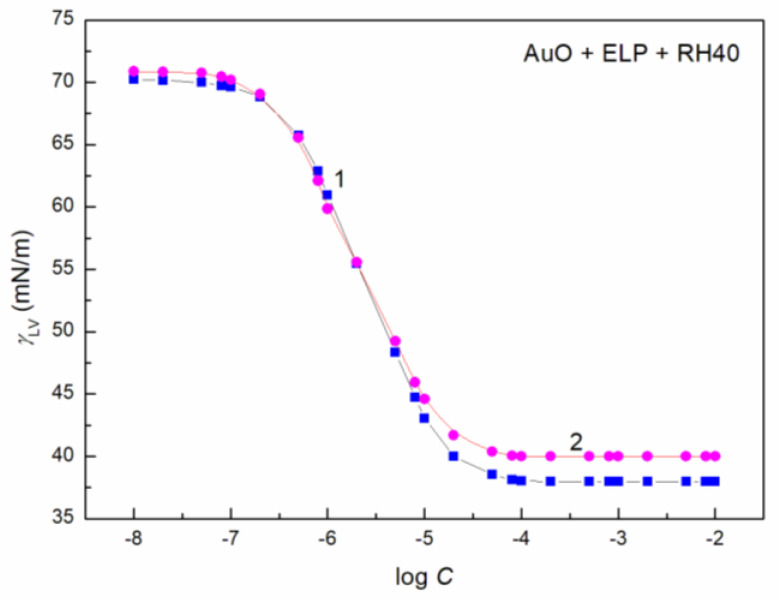
A plot of the surface tension (*γ_LV_*) of aqueous solutions of ELP + RH40 (mole fraction of ELP in the bulk phase, *α*, equal to 0.8) (curve 1, squares) and AuO + ELP + RH40 (*α* = 0.8) mixtures (curve 2, circles) vs. the logarithm of ELP + RH40 concentration (log *C*).

**Figure 5:**
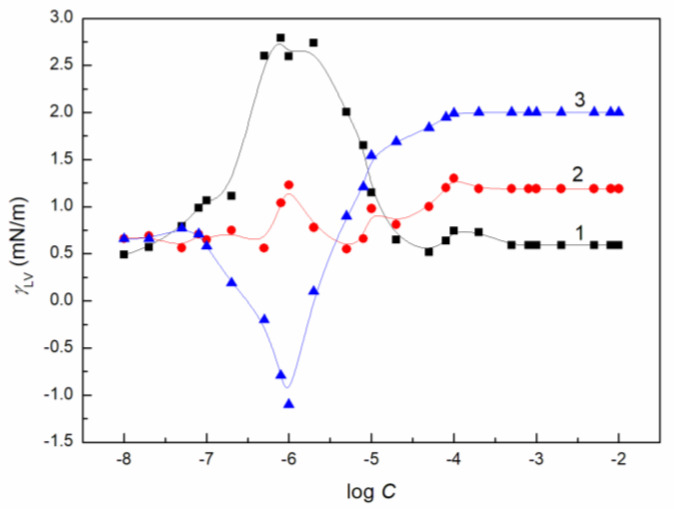
A plot of the difference between the surface tension (*γ_LV_*) of aqueous solutions of AuO + ELP and ELP (curve 1), AuO + RH40 and RH40 (curve 2) and AuO + ELP + RH40 (*α* = 0.8) and ELP + RH40 (*α* = 0.8) (curve 3) vs. the logarithm of ELP, RH40 and ELP + RH40 concentration, respectively.

To study the AuO effect on the surface tension of the aqueous solution of ELP, RH40 and the mixture of ELP and RH40, the concentration of AuO equal to 1 × 10^−5^ mol/dm^3^ was applied. At this AuO concentration, the water surface tension at 293 K is only reduced from 72.8 to 70 mN/m. In the presence of AuO at this concentration, the values of surface tension at almost each concentration of the aqueous solutions of the ELP, RH40 and ELP and RH40 mixture increase (Figure 1, Figure 2, Figure 3, Figure 4 and Figure 5). This is particularly evident in the concentration range of the ELP, RH40 or ELP + RH40 at which the saturated mixed monolayer at the water–air interface is formed. This indicates that in this concentration range of the surfactants, there is a linear dependence between the surface tension and log *C* (*C* is the concentration of surfactant or the surfactants mixture) before critical micelle concentration (CMC). In this concentration range, probably the adsorption of the surfactant molecules together with AuO at the water–air interface takes place. As a result, the surfactants’ concentration in the monolayer at this interface in the AuO presence is smaller than in the AuO absence at the same surfactants’ concentration in the bulk phase. As the AuO surface tension is greater than that of the surfactant tail, the changes of the surfactant molecules on the AuO in the mixed monolayer cause an increase in the solution surface tension. It is interesting that the minimal surface tension of the aqueous solution of ELP or RH40 and/or ELP + RH40 in the presence of AuO is close to the surface tension of solutions without AuO. From the theoretical and practical points of view it is important to describe and/or to predict the isotherm of the surface tension of the aqueous solution of the mixture of different compounds based on the isotherms of the surface tension of particular components of the mixture.

It proved that all obtained isotherms of the surface tension can be described by the exponential function of the second order (Figure 1 and Figures S1–S3 in Supplementary Materials) which has the form:

(5) $$\gamma_{LV}=y^{0}+A_{1}\exp\left(\frac{-C}{t_{1}}\right)+A_{2}\exp\left(\frac{-C}{t_{2}}\right),$$

where *y*_0_, *A*_1_, *A*_2_, t_1_ and t_2_ are the constants.

It is difficult to connect the components and parameters of the surfactants surface tension or AuO with the constants in Equation (5). Nevertheless, it can be suggested that the minimum surface tension of the aqueous solution of the studied surfactants and their mixture in the AuO presence depends on the AuO surface tension and LW components of water and surfactant tail surface tension that should be related to the *y*^0^ value. However, the other constants in Equation (5) should be related to the electron-acceptor and electron-donor parameters of the water, surfactant head and AuO surface tension to a smaller extent. The constants in Equation (5) for the aqueous solution of ELP or RH40 and/or the ELP and RH40 mixture in the AuO presence differ from those for solution without AuO insignificantly. It proves that AuO as the fluorescent probe affects the surface properties of such surfactants as ELP and RH40.

The isotherm of the surface tension of the aqueous solution of AuO, AuO + ELP, AuO + RH40 and AuO + ELP + RH40 can be also described by the Szyszkowski equation (Figure 1 and Figures S1–S3 in Supplementary Materials), which can be expressed as [15]:

(6) $$\gamma_{W}-\gamma_{LV}=\pi =RT\Gamma^{max}\ln\left(\frac{C}{a}+1\right),$$

where *a* is the constant related to the Gibbs free energy of adsorption, Γ*^max^* is the maximal concentration of the surfactants and/or their mixtures in the monolayer at the water–air interface, *R* is the gas constant and *T* is the temperature.

In the calculation of *γ_LV_* it was taken into account that only compounds whose molecules are not aggregated in the bulk phase influence the water surface tension. Indeed Equation (6) was solved numerically choosing the proper values of Γ*^max^* and *a*. The presence of AuO in the aqueous solution of ELP and RH40 as well as their mixture does not change the maximal concentration of the AuO + ELP, AuO + RH40 and AuO + ELP + RH40 mixtures practically in comparison with the single surfactants and/or their mixture in the absence of AuO (Table 1). This suggests that AuO does not influence the total concentration of the mixed monolayer at the water–air interface but changes its composition. This causes that there is the difference between the surface tension of the aqueous solution of surfactants and their mixtures in the presence of AuO and solutions without AuO. In the case of the constant *a*, AuO has an insignificant effect on its value (Table 1). This indicates that AuO influences the ELP, RH40 and ELP + RH40 mixture tendency to adsorb at the water–air interface insignificantly.

In the literature, it is possible to find not only the concepts that enable description of the isotherm of surface tension of the surfactants and their mixtures solution but also those useful for the prediction of the isotherms of surface tensions of multicomponent solutions based on the surface tension isotherms of solutions of individual mixture components [24,25,26]. Among them the concepts proposed by Fainerman and Miller [25,26] for the solutions of binary mixture of the surfactants from the same homological series appeared to be useful for more complicated systems after its modification. The main problem concerning the Fainerman and Miller equation usage for prediction of the isotherms of the surface tension of the aqueous solution of multicomponent mixture was to establish the limiting area occupied by one molecule of a given mixture component at the water–air interface and the average area of the surfactants mixture or molecule at this interface. It was proposed by us that this average area depends on the limiting area of all components of the mixture and fraction of the interface area occupied by a given component [11,19]. It proved that this fraction can be determined based on the pressure of the monolayer of a given components mixture at the water–air interface (*π_i_*).

The Fainerman and Miller equation [25,26] for the aqueous solution of the ternary mixture of compounds can be written in the form:

(7) $$\exp\Pi ={\exp\Pi }_{1}+{\exp\Pi }_{2}+{\exp\Pi }_{3}-2,$$

In the case of the solution of the binary mixture of surfactants Equation (7) can be expressed as:

(8) $$\exp\Pi ={\exp\Pi }_{1}+{\exp\Pi }_{2}-1,$$

where Π=πϖ/RT, $\Pi_{1}=\pi_{1}\varpi_{1}/RT$, $\Pi_{2}=\pi_{2}\varpi_{2}/RT$ and $\Pi_{3}=\pi_{3}\varpi_{3}/RT$ are the dimensionless pressure of the mixed monolayer at the water–air interface and surfactants 1, 2 and 3, respectively, and $\varpi_{1}$, $\varpi_{2}$, $\varpi_{3}$ and ϖ are the areas occupied by one mole of surfactants 1, 2 and 3 and the mixture at the water–air interface.

It appeared that there are some differences between the measured and calculated from Equations (7) and (8) isotherms of the surface tension (Figures S1–S3). Unexpectedly, the greatest agreement between the measured and calculated surface tension isotherms was obtained for the solution of AuO + ELP + RH40 mixture (Figure S3). For the calculation of the isotherms of the surface tension of the studied solutions, it was assumed that the limiting area occupied by the AuO molecule at the interface corresponds to its contactable area at the parallel orientation of its molecule. It is not excluded that the AuO molecule in the mixture with ELP and RH40 can be oriented perpendicularly to the interface. For such a case, the limiting area occupied by one AuO molecule is considerably smaller than at the parallel orientation, and perhaps for this reason there are some differences between the calculated and measured isotherms of adsorption.

If in the mixed monolayer at the water–air interface there are no strong intermolecular interactions, then the surface tension of the aqueous solution of AuO + ELP or AuO + RH40 and/or AuO + ELP + RH40 should satisfy the equations [11,19]:

(9) $$\gamma_{LV}=\gamma_{LV}^{1}X_{1}^{S}+\gamma_{LV}^{2}X_{2}^{S}+\gamma_{LV}^{3}X_{3}^{S},$$

and

(10) $$\gamma_{LV}=\gamma_{LV}^{1}X_{1}^{S}+\gamma_{LV}^{2}X_{2}^{S},$$

where $\gamma_{LV}^{1}$, $\gamma_{LV}^{2}$ and $\gamma_{LV}^{3}$ are the surface tension of the aqueous solution of compounds 1, 2 and 3 at a given concentration in the bulk phase and $X_{1}^{S}$, $X_{2}^{S}$ and $X_{3}^{S}$ are the mole fraction of surfactants 1, 2 and 3 in the mixed monolayer.

For the binary mixture of compounds, it was earlier suggested that $X_{1}^{S}$ and $X_{2}^{S}$ can be determined using the film pressure of surfactants 1 and 2 at the concentration in their individual solutions. Hence, $X_{1}^{S}=\frac{\pi_{1}}{\pi_{1}+\pi_{2}}$ and $X_{2}^{S}=\frac{\pi_{2}}{\pi_{1}+\pi_{2}}$ (*π*_1_ and *π*_2_ are the layers of surfactants 1 and 2 pressure, respectively). In the case of ternary mixture $X_{1}^{S}=\frac{\pi_{1}}{\pi_{1}+\pi_{2}+\pi_{3}}$, $X_{2}^{S}=\frac{\pi_{2}}{\pi_{1}+\pi_{2}+\pi_{3}}$ and $X_{3}^{S}=\frac{\pi_{3}}{\pi_{1}+\pi_{2}+\pi_{3}}$. The calculated isotherms of the surface tension of the aqueous solution of AuO + ELP or AuO + RH40 and/or AuO + ELP + RH40 are slightly different from those measured (Figures S1–S3). This may be related to strong interactions of AuO molecules with the surfactants in the mixed monolayer at the water–air interface.

### 2.3. Concentration and Composition of the Mixed Monolayer at the Water–Air Interface

The interactions of the AuO molecules with those of surfactants should be reflected in the concentration and composition of the mixed monolayer at the water–air interface. As the concentration of AuO in the solution in all the studied systems was the same and constant, and therefore the surface concentration of ELP or RH40 and/or the mixture of ELP with RH40 can be determined using the Gibbs isotherm equation, which has the form [15]:

(11) $$\Gamma =-\frac{C_{i}}{nRT}{\left(\frac{\partial \gamma_{LV}}{\partial C_{i}}\right)}_{C_{j\neq i},T}=-\frac{1}{2.303nRT}{\left(\frac{\partial \gamma_{LV}}{\partial \log C}\right)}_{C_{j\neq i},T},$$

where Γ is the Gibbs surface excess concentration, which for the surfactants is practically equal to their total concentration in the monolayer at the interfaces, *C_i_* is the concentration of the *i* component of the solution and/or the sum of concentration of some components of solution, and *n* depends on the kind of surfactants being equal to unity for the nonionic surfactants. It should be remembered that Equation (11) has some limitations, namely the activity coefficient of *i* component of the solution should be close to unity and $X_{i}≅\frac{C_{i}}{\omega }$, where *ω* is the number of the water moles in 1 dm^3^.

The values of Γ calculated from Equation (11) confirm that AuO influences the adsorption of ELP and RH40 as well as their mixtures (Figures S4–S6). In the case of the ELP and RH40 mixture in the presence of AuO at its constant concentration, it is possible to determine only the sum of ELP and RH40 Gibbs surface excess concentration. Thus, we do not know which component of the surfactant mixtures is susceptible to the action of AuO in the process of its adsorption which is associated with the concentration of the particular components of AuO + ELP + RH40 mixture as well as the mixtures AuO with ELP and/or RH40.

It seems that more information about the influence of AuO on the adsorption of particular studied surfactants can be obtained from the data originated from the Frumkin isotherm adsorption equation modified by us for the surfactant mixtures [15]:

(12) $$\pi_{i}=-RTX_{i}\Gamma_{i}^{max}\ln\left(1-\frac{\Gamma_{i}}{X_{i}\Gamma_{i}^{max}}\right),$$

where *π_i_* is the contribution of *i* component of the mixture to the reduction of water surface tension, $\Gamma_{i}^{max}$ is the maximal concentration of *i* component of the mixture in the monolayer at the water–air interface and *X_i_* is the molar fraction of *i* component of the mixture in the mixed monolayer at the water–air interface. The proposed form of Frumkin equation is based on the assumption that $\pi =\sum_{n}^{n=1}\pi_{i}X_{i}$ and $\Gamma^{max}=\sum_{n}^{n=1}\Gamma_{i}^{max}X_{i}$.

It should be emphasized that on the basis of Equation (12), it is possible to determine only the contribution of a given component of the surfactants’ mixture to the reduction of the water surface tension but not the total contribution of all components of the mixture [27,28]. In this equation, *π_i_* is not equal to the difference between the water surface tension and the aqueous solution of the surfactants mixture but the difference between the water surface tension and the aqueous solution of given single component at the film pressure equal to *π_i_*.

As follows from the calculation of Γ*_i_*, using Equation (12) AuO reduces the RH40 adsorption at the water–air interface to a greater extent than ELP, and the maximal concentration of surfactants and their mixture in the AuO presence is smaller than in its absence (Figures S7–S9). On the other hand, the maximal concentration of the surfactants and their mixtures with AuO determined from the Szyszkowski equation practically does not differ from those without AuO (Table 1). What could it result from? As mentioned above, the result of strong attractive interactions between the AuO molecules with those of ELP and RH40 ones can change the orientation at the water–air interface from parallel to perpendicular toward the water–air interface, and then $\Gamma_{i}^{max}$ is considerably higher than that used in the Frumkin equation. Thus, the AuO contribution to the water surface tension reduction is greater than that obtained from Equation (12). On the other hand, the mole fraction of particular components, particularly in the saturated monolayer, can be different from that determined based on the surface tension isotherms of the aqueous solutions of these components.

The relative mole fraction of particular components of the surfactants mixture can be determined, among others, using the Hua and Rosen concept [15,16]. It was shown earlier that the Hua and Rosen concept proved for binary mixture can be successfully used for the ternary mixture of surfactants. In such case, the binary mixture is treated as one. For our system, the Hua and Rosen equation can be expressed as [15,16]:

(13) $$\frac{{\left(X_{12}\right)}^{2}\ln\left(\alpha_{12}C_{123}/X_{12}C_{12}\right)}{{\left(1-X_{12}\right)}^{2}\ln\left[\left(1-\alpha_{12}\right)C_{123}/\left(1-X_{12}\right)C_{2}\right]}=1,$$

where *X*_12_ = *X*_1_ + *X*_2_ is the summary mole fraction of the AuO and ELP or RH40 in the mixed monolayer at the water–air interface, *α*_12_ = *α*_1_ + *α*_2_ is the summary mole fraction of AuO and ELP or RH40 in the ternary mixture in the bulk phase, *C*_12_ = *C*_1_ + *C*_2_ is the summary mole concentration of AuO and ELP or RH40 in the bulk phase, and *C*_123_ = *C*_1_ + *C*_2_ + *C*_3_ is the summary concentration of the AuO, ELP and RH40 mixture in the bulk phase. The summary mole fraction of the ternary mixture in the mixed monolayer at the water–air interface and in the bulk phase is equal to unity. Hence, *X*_3_ = 1 − *X*_12_ and *α*_3_ = 1 − *α*_12_. To find the mole fractions of AuO, RH40 and ELP in the mixed monolayer Equation (13) was numerically solved assuming firstly that AuO + ELP is the one component and secondly that one component is the sum of AuO and RH40. It should be remembered that the concentration of the component or the sum of components corresponds to the same value of the surface tension of their aqueous solutions.

Based on the obtained composition of the mixed monolayer at the water–air interface using Equation (13) (Table 2), it can be stated that the effect of AuO on the adsorption of the ELP and RH40 is more evident than it results directly from the isotherm of the surface tension of the aqueous solution of AuO. Unfortunately, it is impossible to use Equation (13) for determination of the composition of the AuO + ELP or AuO + RH40 mixtures directly from the isotherms of the surface tension of aqueous solution of AuO and ELP and/or AuO and RH40. However, it can be stated that the AuO effect is greater in the case of ELP adsorption than that of RH40. Knowing the composition of the mixed monolayer at the water–air interface, it is possible to determine the parameter of the intermolecular interactions in the monolayer (*β^σ^*) using the Hua and Rosen concept [15,16]. The equation proposed by them for the *β^σ^* calculation has the form:

(14) $$\beta^{\sigma }=\frac{\ln\left(X_{12}^{b}C_{123}/X_{12}C_{12}\right)}{{\left(1-X_{12}\right)}^{2}},$$

The *β^σ^* values calculated from Equation (14) are negative (Table 2). This means that there are attractive interactions between the molecules in the mixed monolayer and indicates synergetic effect in the water surface tension reduction by the mixed monolayer.

### 2.4. Volumetric Properties

**Figure 6:**
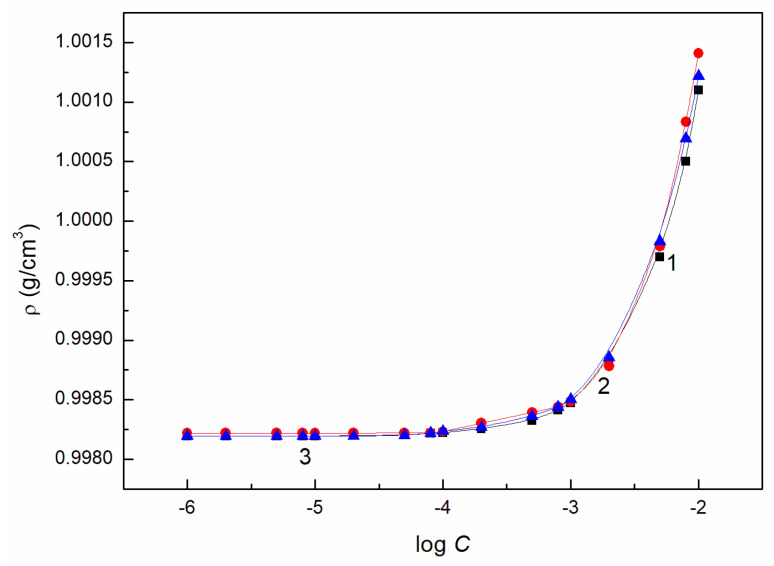
A plot of the density (*ρ*) of the aqueous solutions of AuO + RH40 (curve 1, squares), AuO + ELP (curve 2, circles) and AuO + ELP + RH40 mixtures (curve 3, triangles) vs. the logarithm of RH40, ELP or ELP + RH40 concentration (log *C*).

Due to the above mentioned strong interactions of AuO with ELP and RH40, AuO influences significantly the adsorption properties of these surfactants. The interactions between AuO and the surfactant molecules also affect the volume properties of aqueous surfactant solutions and their mixtures. This fact is confirmed by the density isotherms (Figure 6 and Figures S10–S12). The AuO influence on the density isotherms of ELP, RH40 aqueous solutions and their mixtures is particularly visible in the range of surfactants’ concentration in which they are present in the micellar form in the solution [10,11]. This indicates that AuO has a greater effect on the micellar pseudo phase than on the solution in which the surfactants are present in the monomeric forms. It is possible to determine the average apparent molar volume of AuO and surfactants (*ϕ_V_*) from the density isotherms using the following equation [11]:

(15) $$\phi_{V}=\frac{M_{S}}{{ϼ}_{0}}+\frac{1000\left({ϼ}_{0}-ϼ\right)}{C_{S}{ϼ}_{0}},$$

where *M_S_* is the average molecular weight of AuO and surfactant, $C_{S}$ is the sum of the concentration of AuO and surfactants in mol/cm^3^, and ${ϼ}_{0}$ and ϼ are the density of a “pure” solvent and the solution, respectively.

**Figure 7:**
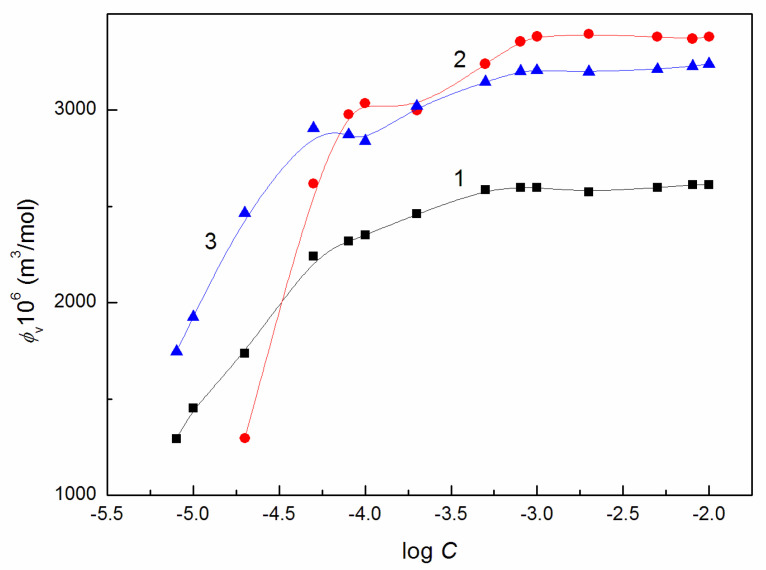
A plot of the apparent molar volume (*ϕ_V_*) of the aqueous solutions of AuO + RH40 (curve 1), AuO + ELP (curve 2) and AuO + ELP + RH40 mixtures (curve 3) vs. the logarithm of RH40, ELP or ELP + RH40 concentration (log *C*).

The calculated values of *ϕ_V_* confirm that AuO influences the volumetric properties of the aqueous solution of ELP, RH40 and their mixture (Figure 7 and Figures S13–S15). In fact, the apparent molar volume of the mixture depends on the molar volume of the components of this mixture. Thus, for the ternary mixture it can be written:

(16) $$\phi_{V}=V_{1}\alpha_{1}+V_{2}\alpha_{2}+V_{3}\alpha_{3},$$

and for the binary mixture:

(17) $$\phi_{V}=V_{1}\alpha_{1}+V_{2}\alpha_{2},$$

where *V* is the molar volume of the component of the mixture and 1, 2 and 3 refer to the particular component of the mixture.

Taking into account the molar volume of AuO, ELP and RH40 calculated based on the bonds’ length between the atoms in the molecule and the angle between them, and the same average distance between the molecules and the mole fraction of the particular compound in the mixture the *ϕ_V_* values were determined from the above-presented equations.

The values of the molar volume of AuO, ELP and RH40 calculated theoretically were equal to 285.2, 2637.53 and 2906.27 cm^3^/mol, respectively. These values are close to those obtained from the density of AuO, ELP and RH40. The values of *ϕ_V_* determined in the discussed way are higher for the RH40 and AuO mixture and lower for the AuO + ELP and AuO + ELP + RH40 mixtures than those determined from Equation (15) (Figures S13–S15). These discrepancies may be due to two reasons. Firstly, the average molar weight is different from those deduced based on the molar weight of particular components of the mixture and the molar fraction of each component in the mixture. Secondly, the distance between the AuO, ELP and RH40 molecules and that of water is different from 2 Å, which was taken into account in the calculation of the molar volume and can be different for the molecules of AuO and the surfactants in the micelle.

The presence of AuO in the micelles can be deduced from the comparison of critical micelle concentration in the presence and absence of AuO in the solution of the surfactants determined using the different methods (Table 3). The presence of AuO in the aqueous solution of surfactants and their mixture causes the decrease in the surfactants’ concentration at which the aggregation process took place. However, the total concentration of AuO and surfactants at which the micellization process proceeds is higher than that of the surfactants without AuO (Table 3). As it was mentioned above, the CMC values are different depending on the method of their determination (Table 3). It is known that the aggregation process occurred rather in the some range of the surfactants’ concentration but not at the precisely determined value of this concentration. Different macroscopic physicochemical properties of the solutions can be more or less sensitive to the microscopic changes in the bulk phase of the solution. Hence, the greatest values of CMC obtained from the fluorescence measurements in the AuO presence in the aqueous solution of surfactants are rather associated not with CMC but with the concentration at which the size and shape of micelles are changed (Table 3). It is worth emphasizing that the CMC values obtained from the fluorescence measurements in the presence of pyrene are similar to those obtained from the surface tension isotherms (Table 3) [11]. This proves that pyrene, unlike AuO, does not affect the micellization process of the investigated surfactants. Differences in the CMC values of surfactants without and in the presence of AuO may suggest significant solubilization of AuO. This conclusion can be confirmed based on the Hua and Rosen theory [16] due to the composition of mixed micelles. As follows from this theory, it is possible to determine the composition of the micelles including the AuO, ELP and RH40 mixture. For this mixture, the modified Hua and Rosen equation can be expressed as [15,16]:

(18) $$\frac{{\left(X_{12}^{M}\right)}^{2}\ln\left(\alpha_{12}C_{123}^{M}/X_{12}^{M}C_{12}^{M}\right)}{{\left(1-X_{12}^{M}\right)}^{2}\ln\left[\left(1-\alpha_{12}\right)C_{123}^{M}/\left(1-X_{12}^{M}\right)C_{3}^{M}\right]}=1,$$

where $X_{12}^{M}=X_{1}^{M}+X_{2}^{M}$ is the summary mole fraction of AuO and ELP or RH40 in the micelle,$\;C_{12}^{M}$, $C_{3}^{M}$ and $C_{123}^{M}$ is the CMC of the AuO+ ELP mixture or AuO + RH40, the CMC of RH40 or ELP and CMC of the AuO + ELP + RH40 mixture, respectively.

The values of the relative mole fraction of AuO, ELP and RH40 calculated from Equation (18) are equal to 0.2115, 0.6021 and 0.1864, respectively. They indicate that the mole fraction of AuO in the micelle is considerably greater than in the monomeric state and that the ELP and RH40 mole fractions are smaller in the micelle than in the monomeric form. The mole fraction of ELP in the micelle is in the greater degree lower than RH40 in comparison to the monomeric state. This indicates that AuO, similarly to the adsorption at the water–air interface, affects the process of ELP micellization to a greater extent than on RH40. The presence of AuO in the micelles indicates the above-mentioned positive interactions between the surfactant and the AuO molecules. This conclusion confirms the parameter of the intermolecular interactions (*β^M^*), which can be determined from the following equation [15,16]:

(19) $$\beta^{M}=\frac{\ln\left(\alpha_{12}C_{123}^{M}/X_{12}^{M}C_{12}^{M}\right)}{{\left(1-X_{12}^{M}\right)}^{2}},$$

Equation (19) was solved for all possible cases and the obtained results show that the *β^M^* parameter is negative and can be in the range of −2.4 to −1.5. This confirms that there are positive interactions of AuO with surfactants.

The calculations of the mole fraction of particular components of the AuO + ELP + RH40 mixture in the micelles as well as the parameter of intermolecular interactions in the micelle indicate the AuO solubilization process. Thus, this process was analyzed in detail based on the fluorescence measurements of the aqueous AuO + ELP solutions at different temperatures. Based on the obtained results, the changes of CMC of ELP as a function of temperature [11], the thermodynamic parameters of the micellization as well as the solubilization were considered. The obtained values of CMC for the AuO + ELP mixture (Table 3), as mentioned above, are considerably higher than those determined by other methods as well as than CMC for ELP in the absence of AuO [11]. However, the course of changes in CMC as a function of temperature for the AuO + ELP mixture is similar to the changes in CMC without the presence of AuO [11].

As mentioned above, the comparison of the CMC values of the AuO + ELP mixture with those for ELP without AuO suggest great solubility of AuO in the ELP micelles. This suggestion can be confirmed by the parameters of the solubilization process. These parameters were considered using two models, mainly the model based on the mass action law and the Nernst law of partition [29,30]. According to the mass action law: $AuO_{f}+M\;\overset{K_{b}}{\Leftrightarrow }AuO_{b}$, where $K_{b}=\frac{{\left[AuO\right]}_{b}}{{\left[AuO\right]}_{f}\cdot \left[M\right]}$ is the binding constant of AuO molecule with micelle [*AuO*]*_b_*—the concentration of AuO associated with the surfactant micelles in relation to the total solution volume; [*AuO*]*_f_*—the free AuO concentration with regard to the total volume of the solution; [*M*]—the concentration of surfactant micelles in relation to the total volume of the solution.

For the constant concentration of the AuO in all the studied solutions ([*AuO*]_0_), the intensity of the fluorescence at the wavelength of the light emitted *λ* changes with the total concentration of surfactant (*C_S_*_,0_) in the solution according to the relationship [30,31]:

(20) $$F\left(\lambda ,C_{S,0}\right)=\frac{F_{f}\left(\lambda \right)+F_{b}\left(\lambda \right)K_{b}\cdot \left[M\right]}{1+K_{b}\cdot \left[M\right]}=\frac{F_{f}\left(\lambda \right)+F_{b}\left(\lambda \right)\cdot \frac{K_{b}}{n}\cdot C_{S,M}\left(C_{S,0}\right)}{1+\frac{K_{b}}{n}\cdot C_{S,M}\left(C_{S,0}\right)},$$

where *F_f_*(*λ*), *F_b_*(*λ*) is the fluorescence intensity of “free” AuO (in the aqueous solution) and associated with the micelle at a total concentration equal to [*AuO*]_0_ for the wavelength *λ*, *n* is the surfactant aggregation number. It was assumed that there are only micelles with the aggregation number *n* in the system or that the micelles are polydisperse with the average aggregation number *n*, but the binding constant of the solubilizate with each individual micelle does not depend on its aggregation number and is always $K_{b,}$ and *C_S_*_,*M*_(*C_S_*_,0_) is the dependence of the surfactant concentration in the micellar form on the total surfactant concentration in the system—calculated here in accordance with the W. Al-Soufi, L. Pi’neiro and M. Novo (APN) model (two empirical parameters: CMC and *r*) [30,31].

According to the pseudophase model based on the Nernst partition law, the values of the micelle–water phase partition constant *K_MW_* were determined. *K_MW_* is connected with the expression $AuO_{W}\overset{K_{MW}}{\Leftrightarrow }AuO_{M}$, where $K_{MW}=\frac{{\left[AuO\right]}_{M}}{{\left[AuO\right]}_{W}}$ is the micelle–water phase partition coefficient, ([*AuO*]*_M_* is the concentration of AuO in the micellar pseudophase in relation to the volume of the micellar pseudophase, and ([*AuO*]*_W_* is the concentration of AuO in the aqueous phase (relative to the volume of the aqueous phase).

At the constant concentration of AuO in all samples ([*AuO*]_0_), the fluorescence intensity with the emitted light wavelength *λ* changes with the total concentration of surfactant *C_S_*_,0_ in the solution according to the relationship [32]:

(21) $$F\left(\lambda ,C_{S,0}\right)=F_{W}\left(\lambda \right)+\left(F_{M}\left(\lambda \right)-F_{W}\left(\lambda \right)\right)\cdot \frac{K_{MW}\cdot V_{S,M}^{m}\cdot C_{S,M}\left(C_{S,0}\right)}{1+\left(K_{MW}-1\right)\cdot V_{S,M}^{m}\cdot C_{S,M}\left(C_{S,0}\right)},$$

where *F_W_*(*λ*), *F_M_*(*λ*) is AuO fluorescence intensity in the aqueous solution and in the micellar pseudophase for the wavelength *λ* in relation to the total volume of the system, and $V_{S,M}^{m}$ is the apparent molar volume of the surfactant in the micellar form (assuming that the apparent volume does not depend on the surfactant concentration in the system and neglecting the effect of solubilizate in the micellar pseudophase on its value, it is possible to calculate the volume of the micellar pseudophase per unit volume of surfactant solution for a given concentration of surfactant in the micellar form).

Equations (20) and (21) were solved against a given magnitude numerically. For the calculation using Equations (20) and (21), we took into account the fitted values of *F_f_*(*λ*), *F_b_*(*λ*) (Equation (20)) or *F_W_*(*λ*), *F_M_*(*λ*) (Equation (21)), the values of $\frac{K_{b}}{n}$ (for *n* = 1) (Equation (20)) or *K_MW_* (Equation (21)) as well as the CMC and apparent molar volume values for ELP (Equation (21)) taken from the literature [11]. The fluorescence intensity was measured for *λ* in the range of 455 to 655 nm at an interval equal to 5 nm. To determine the values of *K_b_*, the fitted and established values of CMC and *r* based on Equation (20) and *K_MW_*, CMC and *r* from Equation (21) at the different temperature were used. Details of the performed calculations are included in Supplementary Materials.

Based on the values of the *K_MW_* partition constants calculated in presented ways (defined as the ratio of molar concentrations in relation to the volume of individual phases), the values of the partition constants *K_x_* (defined as the ratio of molar fractions of the solubilizate in both phases) can be calculated [29]:

(22) $$K_{MW}≅K_{x}\cdot \frac{V_{S,M}^{m}}{V_{H2O}^{m}}\;,$$

In turn, based on the *K_MW_* value, the standard Gibbs free energy (Δ*G*^0^) of the transfer of AuO from the water phase to the micellar pseudophase can be calculated:

(23) $$\Delta G^{0}=-RT\ln\;K_{x},$$

Knowing the values of Δ*G*^0^ at different temperatures, it was possible to determine the standard enthalpy (*H*^0^) and entropy (Δ*S*^0^) using the van’t Hoff isotherm equation [33]:

(24) $$\ln K_{x}=-\frac{\Delta H^{0}}{RT}+\frac{\Delta S^{0}}{R},$$

The calculated values of the thermodynamic parameters of the solubilization of AuO molecules are presented in Table 4. As follows from this table, the solubilization process of AuO is spontaneous. This means that there are strong interactions between the molecules of ELP and AuO. This is in agreement with the above-presented conclusions.

### 2.5. Standard Gibbs Free Energy of Adsorption and Micellization

In the discussion presented above, it was stated that AuO influences the concentration and composition of the mixed monolayer as well as on the CMC and the composition of the micelles. The adsorption and micellization process of surfactants is connected with the standard Gibbs free energy of adsorption ($\Delta G_{ads}^{0})$ and micellization ($\Delta G_{mic}^{0}$), respectively. The literature reports many methods, which can be used for these energies’ determination [15]. Among them, the Langmuir method modified by de Boer seems to be useful for mixtures if we know the adsorption isotherms for the individual components of the mixture. According to this method $\Delta G_{ads}^{0}$ can be calculated from the following equation [15]:

(25) $$\frac{A_{i}^{0}}{A_{i}-A_{i}^{0}}\exp\frac{A_{i}^{0}}{A_{i}-A_{i}^{0}}=\frac{C_{i}}{\omega }\exp\left(\frac{-\Delta G_{ads}^{0}}{RT}\right),$$

where $A_{i}^{0}$ is the limiting area occupied by a molecule of surfactant in the surface layer, and $A_{i}=\frac{1}{\Gamma_{i}N}$ is the area occupied by one molecule of surfactant in the monolayer at the interface (*N* is the Avogadro number).

The values of $\Delta G_{ads}^{0}$ calculated from Equation (25) suggest that the tendencies to adsorb AuO + ELP, AuO + RH40 and AuO + ELP + RH40 at the water–air interface are almost the same (Table 1). Indeed, the $\Delta G_{ads}^{0}$ values were assumed to have the constant minimal values corresponding to low concentration of surfactant range. This means that in this concentration range there are no interactions between the surfactant molecules in the mixed surface layer at the water–air interface. From the comparison of the $\Delta G_{ads}^{0}$ values obtained for the surfactants and their mixtures without AuO, it results that AuO does not influence the surfactants’ tendency to adsorb at the water–air interface (Table 1). However, as the concentration of surfactants and their mixture increases, the influence of intermolecular interactions in the mixed monolayer on the $\Delta G_{ads}^{0}$ values appears. These interactions increase with the increasing surfactants’ concentration depending on the surfactants’ type. The tendency of AuO to adsorb deduced from Equation (25) is smaller than that of the surfactants (Table 1).

The $\Delta G_{ads}^{0}$ values were also determined using the constant *a* from the Szyszkowski equation. This constant is related to $\Delta G_{ads}^{0}$ by the equation [11,15]:

(26) $$a=\omega \exp\frac{\Delta G_{ads}^{0}}{RT},$$

where *ω* is the number of the water moles in one dm^3^.

The calculated values of $\Delta G_{ads}^{0}$ from Equation (26) differ only insignificantly from those determined from Equation (25) except for of AuO. The calculations of $\Delta G_{ads}^{0}$ suggest that AuO does not affect the $\Delta G_{ads}^{0}$ of the surfactants but confirms that AuO influences the intermolecular interactions of the surfactant molecules, particularly in the saturated mixed monolayer at the water–air interface.

To prove the influence of AuO on the tendency of ELP, RH40 and their mixture to form the micelles, the $\Delta G_{mic}^{0}$ values were calculated from equation [11]:

(27) $$\Delta G_{mic}^{0}=RT\ln\frac{\mathrm{CMC}}{\omega },$$

Similarly to standard Gibbs free energy of adsorption, the values of $\Delta G_{mic}^{0}$ indicate that the presence of AuO does not affect the tendency to aggregate the surfactants and their mixture (Table 5).

## 3. Materials and Methods

Kolliphor^®^ ELP (ELP) (Cremophor^®^ELP, Polyoxyl 35 Hydrogenated Castor oil, Polyoxyl-35 castor oil), Kolliphor^®^ RH 40 (RH40) (Cremophor^®^ RH 40, Macrogolglycerol hydroxystearate, PEG-40 castor oil, Polyoxyl 40 hydrogenated castor oil) and Auramine O were supplied by Sigma-Aldrich (St. Louis, MO, USA)and used without further purification. The doubly distilled and deionized water used for preparation of the aqueous solutions of individual surfactants with AuO (AuO + ELP and AuO + RH40), and ELP + RH40 mixture (the mole fraction of ELP (α) in the bulk phase equal to 0.8) with AuO (AuO + ELP + RH40) was obtained from the Destamat Bi18E distiller (Inkom Instruments, Warsaw, Poland). The surfactant solution concentration was from 1 × 10^−6^ to 1 × 10^−2^ M and the AuO concentration in the surfactant solutions was equal to 1 × 10^−5^ M.

The surface tension (*γ_LV_*) measurements of the aqueous solution of the AuO + ELP, AuO + RH40 and AuO + ELP + RH40 mixtures were made at the temperature 293 K using the Krüss K100 tensiometer according to the platinum ring tensiometer method (du Nouy’s method) calibrated before the measurements. The calibration was made at 293 K using water and methanol whose surface tension at this temperature was equal to 72.8 and 22.5 mN/m, respectively. The surface tension measurements for each concentration and composition of the studied solutions were repeated at least ten times. The standard deviation of the results obtained from the measurements was ±0.1 mN/m and the uncertainty was in the range from 0.3% to 0.9%.

The stock AuO solution was used for preparation of layers on the polytetrafluoroethylene (PTFE) surface. First, the PTFE plates were washed with a nonionic detergent and next with methanol. Then they were placed twice in an ultrasonic bath in the Milli-Q water for 15 min. Then the plates were dried with warm air for 10 min. Purity of the plates was controlled by the measurement of the water contact angle. The AuO layers were prepared by immersing PTFE in the AuO stock solution for 24 h. For the advancing contact angle (*θ*) measurements on the obtained layers water (Destamat Bi18E), formamide (>99.5%, Sigma-Aldrich (St. Louis, MO, USA) and diiodomethane (>99%, Sigma-Aldrich, St. Louis, MO, USA) were used.

The density of the studied solutions was measured with the U-tube densitometer (DMA 5000 Anton Paar) at the temperature equal to 293 K. The precision of the density and temperature measurements given by the manufacturer is ±0.000005 g cm^−3^ and ±0.001 K. The uncertainty was calculated to be equal to 0.01%. The densitometer was calibrated regularly with distilled and deionized water.

Steady state fluorescence measurements for AuO + ELP at different temperatures and for pyrene (Py) (Sigma-Aldrich, St. Louis, MO, USA) with RH40 and ELP + RH40 mixture at T = 293 K were made using a Hitachi F-2700 Fluorescence Spectrometer with a AuO (C = 1 × 10^−5^ M) and Py (C = 2 × 10^−6^ M) as luminescence probes. Fluorescence excitation was done at 445 nm for AuO and 335 nm for Py, and the emission spectra were recorded in the range of 350–650 nm at the scan speed of 300 nm/min. The excitation and emission slit widths were 2.5 nm.

## 4. Conclusions

The experimental data as well as their considerations allow us to draw many conclusions about the properties of AuO and its influence on the adsorption and micellization of ELP, RH40 and their mixture.

Entering the appropriate parts of the molecule into the cubes with the sizes resulting from the length of chemical bonds, the angle between them and the distance between the molecules, the volume of the AuO molecule can be successfully determined.

The surface tension of AuO results mainly from the Lifshitz–van der Waals intermolecular interactions and is comparable to the surface tension of some sugars. Despite the significant absolute value of the Gibbs free energy of the AuO adsorption at the water–air interface, AuO is a weak agent of water surface reduction.

The presence of ELP and RH 40 in the aqueous solution increases the AuO adsorption at the water–air interface significantly, probably due to the strong hydrophobic interactions between the AuO molecules and tails of the surfactant molecules.

The solubilization of AuO in the ELP and RH40 micelles takes place, which influences the CMC values.

The parameters of the AuO solubilization and the critical micelle concentrations of the surfactant and surfactant mixtures as well as the thermodynamic parameters of the solubilization were determined.

## Figures and Tables

**Table 1:** The values of the *y*_0_, *A*_1_, *A*_2_, t_1_, t_2_ (Equation (5)), Γ*^max^* (mol/m^2^, Equation (6)), *a* (Equation (6)) and $\Delta G_{ads}^{0}$ (kJ/mol, Equation (24)) for AuO, ELP and RH40 as well as AuO + ELP, AuO + RH40 and AuO + ELP + RH40 mixtures.

	RH40	ELP	ELP + RH40	AuO	AuO + RH40	AuO + ELP	AuO + ELP + RH40
*y*_0_	41.2097	39.8451	38.0544	64.8984	42.5108	39.9573	40.1669
*A*_1_	16.933	10.2290	15.5921	4.4116	16.8497	19.8769	14.8602
t_1_	9.8449 × 10^−7^	6.3713 × 10^−7^	9.2388 × 10^−6^	1.3 × 10^−4^	1.0369 × 10^−6^	7.7851 × 10^−6^	1.0991 × 10^−6^
*A*_2_	13.5446	22.5086	17.0020	2.9225	12.9216	12.3073	16.4526
t_2_	1 × 10^−5^	7.7375 × 10^−6^	1.6519 × 10^−6^	8.547 × 10^−6^	1.3725 × 10^−5^	1.2070 × 10^−6^	7.8331 × 10^−6^
Γ*^max^* × 10^−6^	2.8	3.1	3.1	0.54	2.8	3.1	3.1
*a*	2.3 × 10^−7^	3.1 × 10^−7^	2.5 × 10^−7^	1.27 × 10^−6^	2.91 × 10^−7^	2.91 × 10^−7^	2.1 × 10^−7^
$\Delta G_{ads}^{0}$	−47.02	−46.29	−46.81	−42.85	−46.44	−46.44	−47.24

**Table 2:** The values of the mole fraction of the AuO, ELP and RH40 in the mixed monolayer at the water–air interface (***X*** as well as the parameter of the intermolecular interactions (*β^σ^*) for AuO + ELP + RH40 mixture at the different values of surface tension, *γ_LV_*.

*γ_LV_* (mN/m)	*X* AuO	*X* ELP	*X* RH40	*β^σ^* (AuO + ELP) − RH40	*β^σ^* (AuO + RH40) − ELP
65	0.7814	0.0372	0.1814	−4.2225	−0.1257
60	0.6656	0.1548	0.1796	−3.3043	−0.9555
55	0.5814	0.2466	0.172	−2.3371	−0.6928
50	0.3933	0.4180	0.1887	−2.2694	−1.3480
45	0.2525	0.5699	0.1776	−1.9867	−1.9653

**Table 3:** The values of the CMC (mol/dm^3^) of AuO, ELP, RH40 and their mixtures determined from the surface tension measurements (CMC1), AuO emission spectra (CMC2), pyrene emission spectra (CMC3) and density measurements (CMC4). The values of CMC1 for ELP, RH40 and ELP + RH40 are taken from Ref. [10] and CMC2 for ELP from Ref. [11].

	ELP	AuO + ELP	RH40	AuO + RH40	ELP + RH40	AuO + ELP + RH40
CMC1	2.14 × 10^−5^	2.20 × 10^−5^	6.64 × 10^−5^	3.86 × 10^−5^	1.92 × 10^−5^	1.90 × 10^−5^
CMC2	-	2.91 × 10^−4^	-	4.89 × 10^−4^	-	3.25 × 10^−4^
CMC3	1.05 × 10^−5^	-	3.01 × 10^−5^	-	2.05 × 10^−5^	-
CMC4	-	6.94 × 10^−5^	-	9.54 × 10^−6^	-	4.21 × 10^−5^

**Table 4:** The values of *K_MW_*, $V_{S,M}^{m}$, $V_{H_{2}O}^{m}$, *K_x_*, Δ*G*^0^, Δ*H*^0^ and Δ*S*^0^ for AuO in ELP solutions.

T [K]	*K_MW_* [-]	$V_{S,M}^{m}[{\mathrm{cm}}^{3}/\mathrm{mol}]$	$V_{H_{2}O}^{m}[{\mathrm{cm}}^{3}/\mathrm{mol}]$	*K_x_* [-]	Δ*G*^0^ [kJ/mol]
293	119.6 ± 0.8	3706.54	18.048	24,555	−24.64
298	103.0 ± 0.6	3710.84	18.068	21,153	−24.69
303	102.2 ± 0.5	3716.04	18.093	20,984	−25.08
308	92.4 ± 0.6	3722.06	18.123	18,973	−25.24
313	86.3 ± 0.5	3728.87	18.156	17,733	−25.47
318	81.1 ± 0.8	3736.39	18.193	16,655	−25.71

Δ*H*^0^ = −11.41 kJ/mol; Δ*S*^0^ = 0.045 kJ/molK.

**Table 5:** The values of $\Delta G_{mic}^{0}\;$ (kJ/mol) of AuO, ELP, RH40 and their mixtures determined on the basis of CMC1 values (Table 3).

	ELP	AuO + ELP	RH40	AuO + RH40	ELP + RH40	AuO + ELP + RH40
$\Delta G_{mic}^{0}$	−35.98	−35.00	−33.21	−33.98	−36.24	−35.24

## Data Availability

Not applicable.

## Supplementary Materials

The following supporting information can be downloaded at: https://www.mdpi.com/article/10.3390/molecules27238493/s1, Figures S1–S3: A plot of the surface tension (*γ_LV_*) of aqueous solutions of AuO + ELP, AuO + RH40 and AuO + ELP + RH40 mixtures vs. the logarithm of concentration (log *C*); Figures S4–S6: A plot of the Gibbs surface excess concentration (Γ) calculated from Equation (11) for aqueous solutions of AuO + ELP, AuO + RH40 and AuO + ELP + RH40 mixtures vs. the logarithm of concentration (log *C*); Figures S7–S9: A plot of the surface concentration (Γ) calculated from Equation (12) for aqueous solutions of AuO, ELP and RH40 vs. the logarithm of concentration (log *C*); Figures S10–S12: A plot of the density (*ρ*) of aqueous solutions of AuO + ELP, AuO + RH40 and AuO + ELP + RH40 mixtures vs. the logarithm of concentration (log *C*); Figures S13–S15: A plot of the apparent molar volume (*ϕ_V_*) of aqueous solutions of AuO, ELP and RH40 as well as AuO + ELP, AuO + RH40 and AuO + ELP + RH40 mixtures vs. the logarithm of concentration (log *C*); Figure S16: Matched values *F_f_*(*λ*), *F_b_*(*λ*) of fluorescence spectra of AuO in ELP solutions (*r* from 0 to 1); Figure S17: Fitting of the theoretical curves of the fluorescence intensity of AuO in ELP solutions to the experimental data (T = 293 K, *r* from 0 to 1); Figure S18: A plot of the visual variability the fluorescence intensity of AuO in ELP solutions vs. ELP concentration (T = 293 K); Figure S19: Matched values *F_f_*(*λ*), *F_b_*(*λ*) of fluorescence spectra of AuO in ELP solutions (*r* = 10^−6^); Figure S20: Fitting of the theoretical curves of the fluorescence intensity of AuO in ELP solutions to the experimental data (T = 293 K, (*r* = 10^−6^); Figure S21: Matched values *F_f_*(*λ*), *F_b_*(*λ*) of fluorescence spectra of AuO in ELP solutions (established parameters: $V_{S,M}^{m}$, CMC, *r* = 10^−6^); Tables S1–S8: The values of *K_b_* and *K_MW_* calculated at different matched and established parameters.

## Sample Availability

Samples of the compounds are not available from the authors.
