# Supplementary material for: Behavior of Auramine O in the Aqueous Solution of Two Kolliphors and Their Mixture
Source: Molecules. 2022 Dec 2;27(23):8493. doi: 10.3390/molecules27238493 (PMC9740790; doi:10.3390/molecules27238493)
Supplement: Supplementary file 1 [file molecules-27-08493-s001.zip › molecules-2028255-supplementary.pdf]

Supplementary Materials

Behavior of Auramine O in the Aqueous Solution of Two Kolliphors and Their Mixture

Katarzyna Szymczyk, Andrzej Lewandowski, Anna Zdziennicka, Magdalena Szaniawska and Bronisław Jańczuk *

Department of Interfacial Phenomena, Institute of Chemical Sciences, Faculty of Chemistry, Maria Curie-Skłodowska University in Lublin, Maria Curie-Skłodowska Sq. 3, 20-031 Lublin, Poland

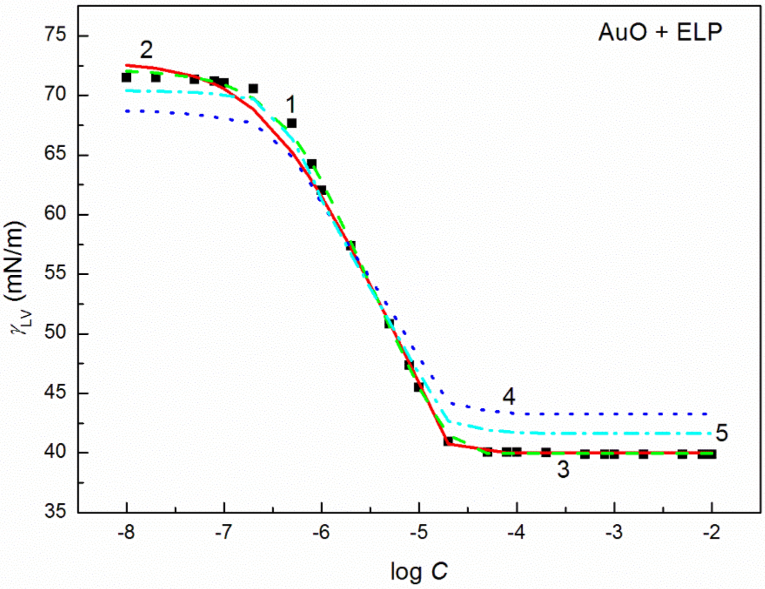

Figure S1. A plot of the surface tension (γ_{LV}) of aqueous solutions of AuO + ELP mixture vs. the logarithm of ELP concentration (log *C*). Points 1 correspond to the measured values, curves 2 – 5 correspond to the values calculated from Eqs. (6), (5), (8) and (10), respectively.

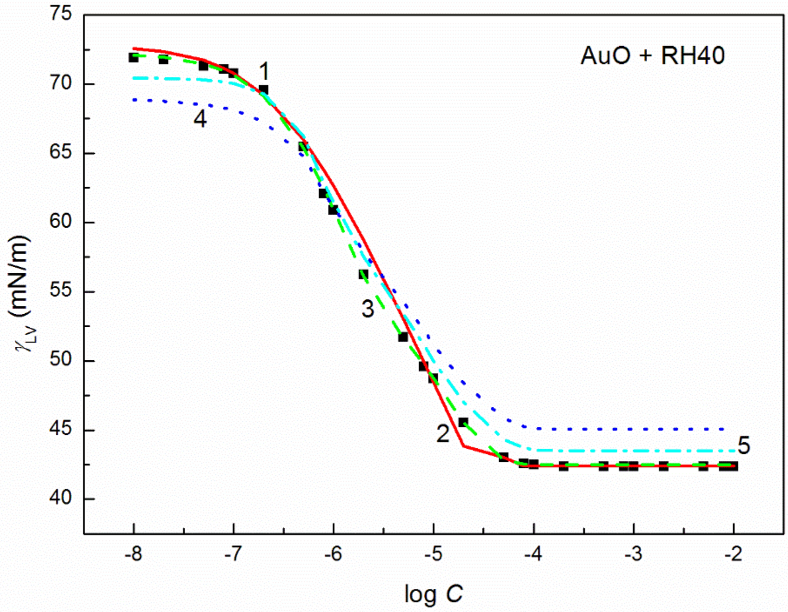

Figure S2. A plot of the surface tension (γ_{LV}) of aqueous solutions of AuO + RH40 mixture vs. the logarithm of RH40 concentration (log *C*). Points 1 correspond to the measured values, curves 2 – 5 correspond to the values calculated from Equations (6), (5), (8) and (10), respectively.

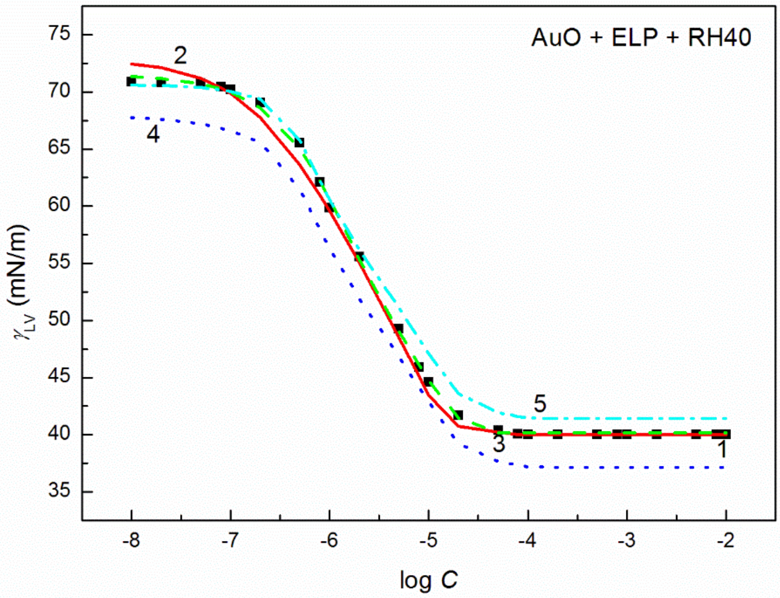

Figure S3. A plot of the surface tension (γ_{LV}) of aqueous solutions of AuO + ELP +RH40 mixture vs. the logarithm of ELP + RH40 concentration (log *C*). Points 1 correspond to the measured values, curves 2 – 5 correspond to the values calculated from Equations (6), (5), (7) and (9), respectively.

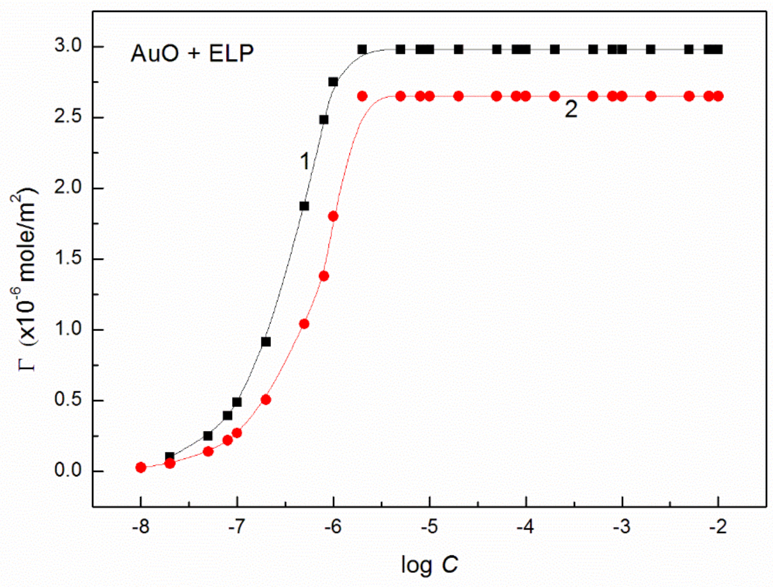

Figure S4. A plot of the Gibbs surface excess concentration (Γ) calculated from Equations (11) for aqueous solutions of ELP (curve 1) and AuO + ELP mixture (curve 2) vs. the logarithm of ELP concentration (log *C*).

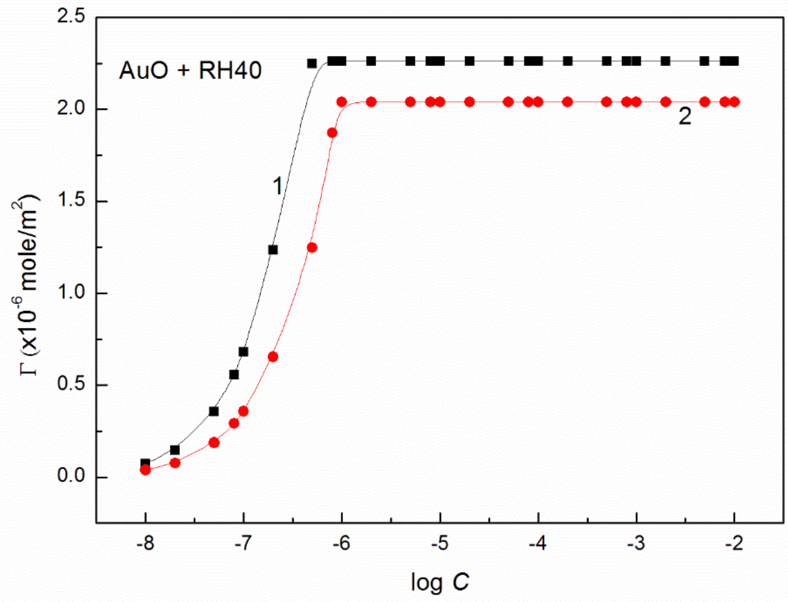

Figure S5. A plot of the Gibbs surface excess concentration (Γ) calculated from Equation (11) for aqueous solutions of RH40 (curve 1) and AuO + RH40 mixture (curve 2) vs. the logarithm of RH40 concentration (log *C*).

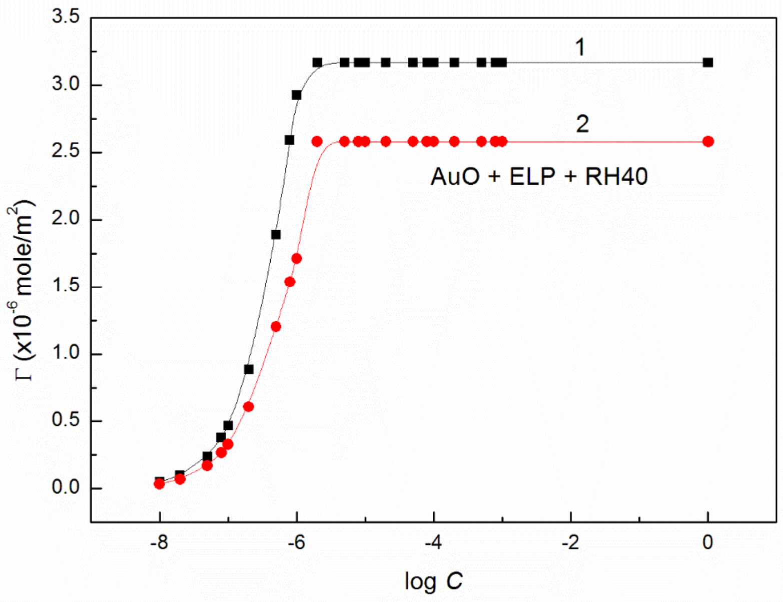

Figure S6. A plot of the Gibbs surface excess concentration (Γ) calculated from Eq. (11) for aqueous solutions of ELP + RH40 (curve 1) and AuO + ELP + RH40 mixture (curve 2) vs. the logarithm of ELP + RH40 concentration (log *C*).

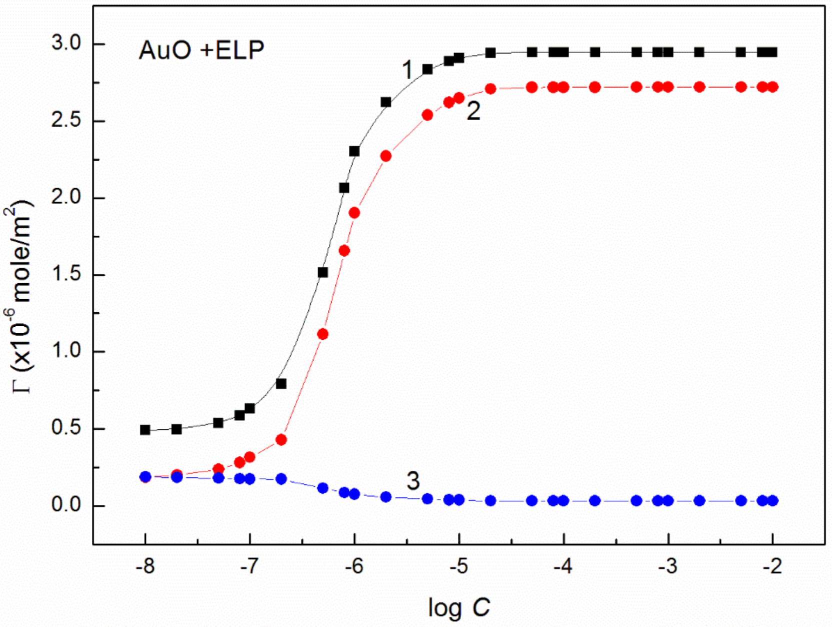

Figure S7. A plot of the surface concentration (Γ) calculated from Eq. (12) for aqueous solutions of ELP (curve 1) as well as ELP (curve 2) and AuO (curve 3) in the AuO + ELP mixture vs. the logarithm of ELP concentration (log *C*).

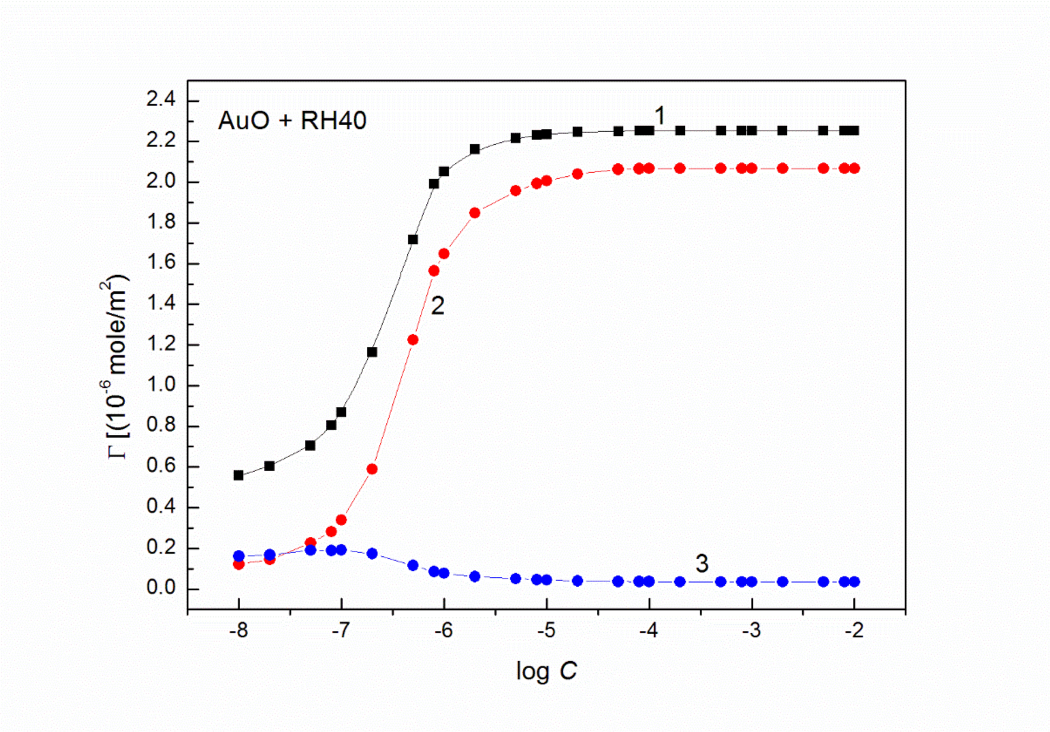

Figure S8. A plot of the surface concentration (Γ) calculated from Eq. (12) for aqueous solutions of RH40 (curve 1) as well as RH40 (curve 2) and AuO (curve 3) in the AuO + RH40 mixture vs. the logarithm of RH40 concentration (log *C*).

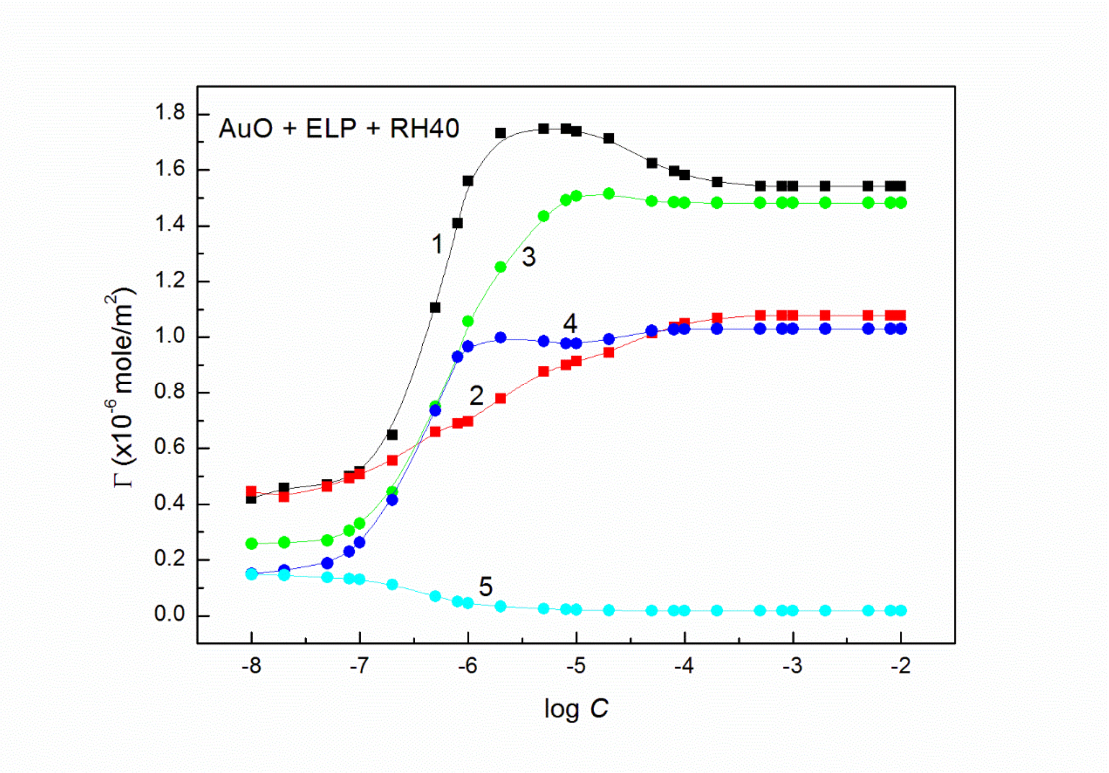

Figure S9. A plot of the surface concentration (Γ) calculated from Eq. (12) for ELP (curve 1) and RH40 (curve 2) in the aqueous solutions of the ELP + AuO mixture as well as for ELP (curve 3), RH40 (curve 4) and AuO (curve 5) in the AuO + ELP + RH40 mixture vs. the logarithm of ELP + RH40 concentration (log *C*).

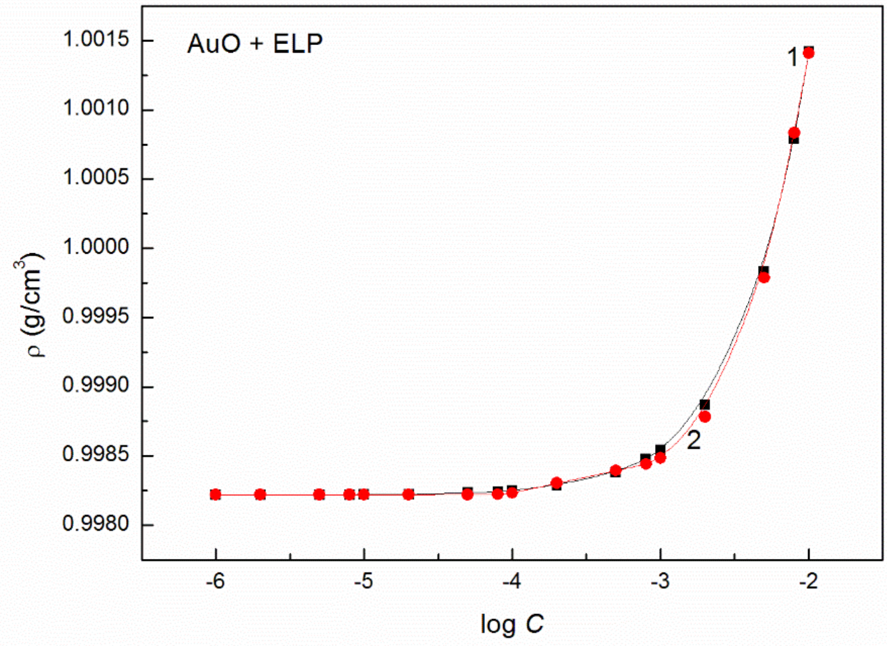

Figure S10. A plot of the density (ρ) of aqueous solutions of ELP (curve 1) and AuO + ELP (curve 2) vs. the logarithm of ELP concentration (log C).

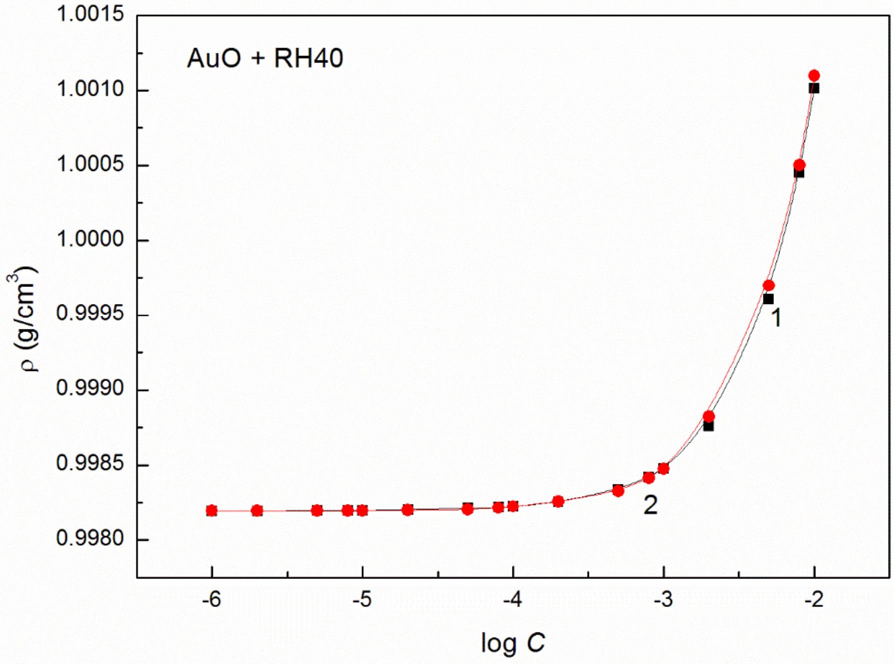

Figure S11. A plot of the density (ρ) of aqueous solutions of RH40 (curve 1) and AuO + RH40 (curve 2) vs. the logarithm of RH40 concentration (log C).

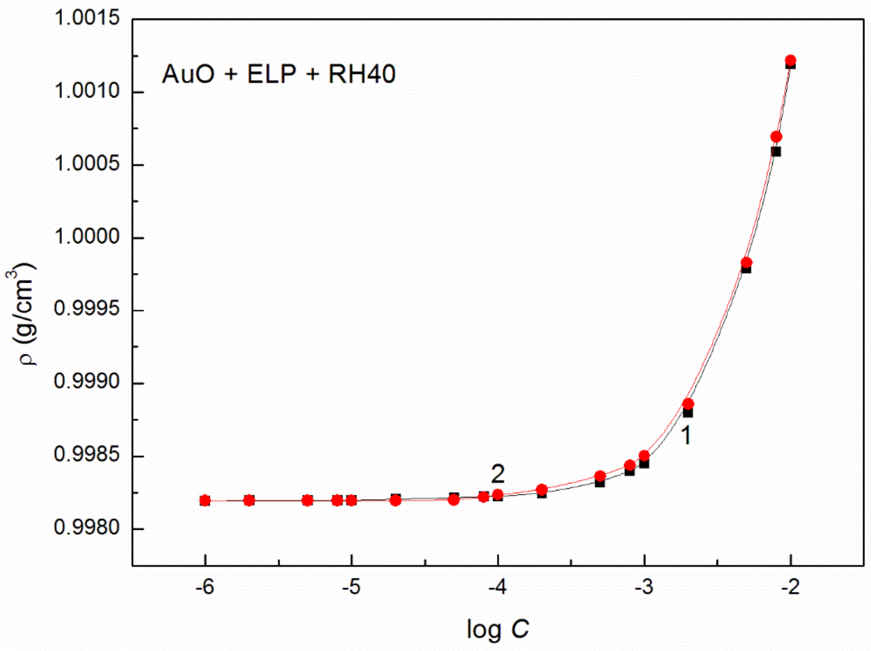

Figure S12. A plot of the density (ρ) of aqueous solutions of ELP + RH40 (curve 1) and AuO + ELP + RH40 mixtures (curve 2) vs. the logarithm of ELP + RH40 concentration (log *C*).

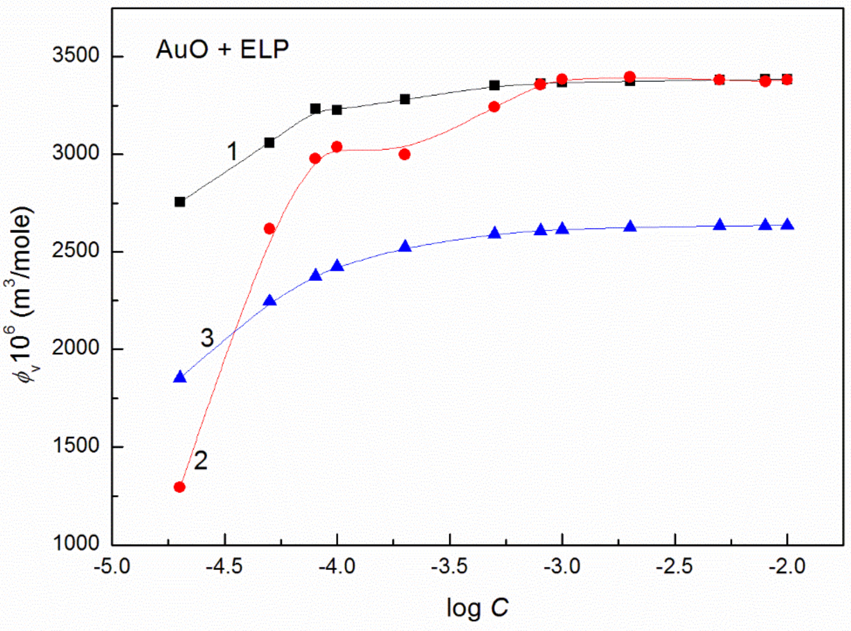

Figure S13. A plot of the apparent molar volume (ϕ_V) of aqueous solutions of ELP (curve 1), AuO + ELP mixture calculated from Eq. (15) (curve 2) and AuO + ELP mixture calculated from Eq. (17) (curves 3) vs. the logarithm of ELP concentration (log *C*).

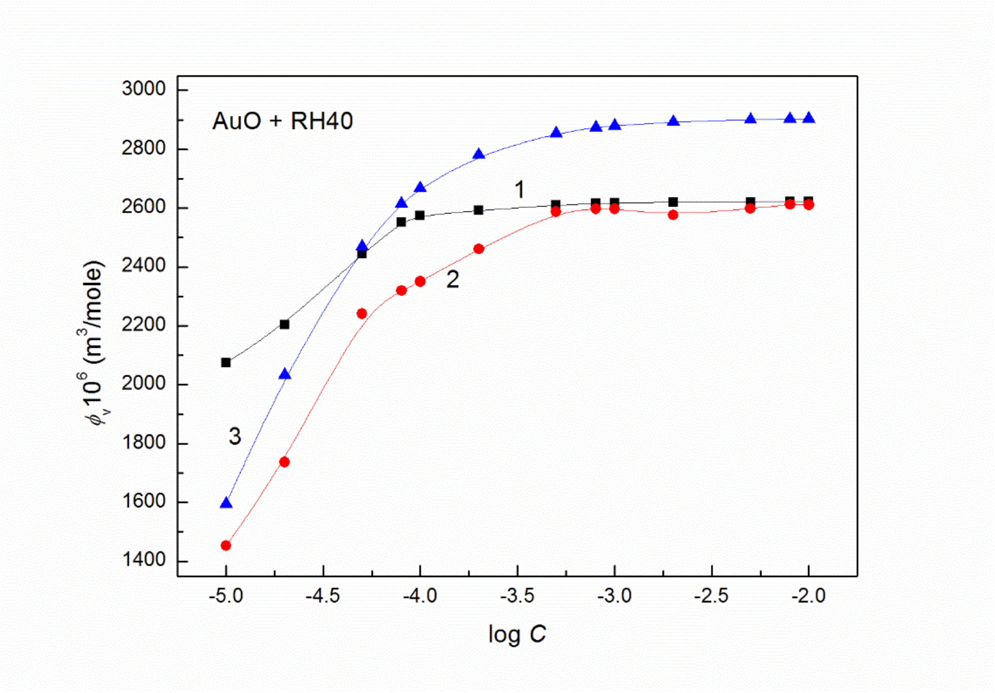

Figure S14. A plot of the apparent molar volume (ϕ_V) of aqueous solutions of RH40 (curve 1), AuO + RH40 mixture calculated from Eq. (15) (curve 2) and AuO + RH40 mixture calculated from Eq. (17) (curves 3) vs. the logarithm of RH40 concentration (log *C*).

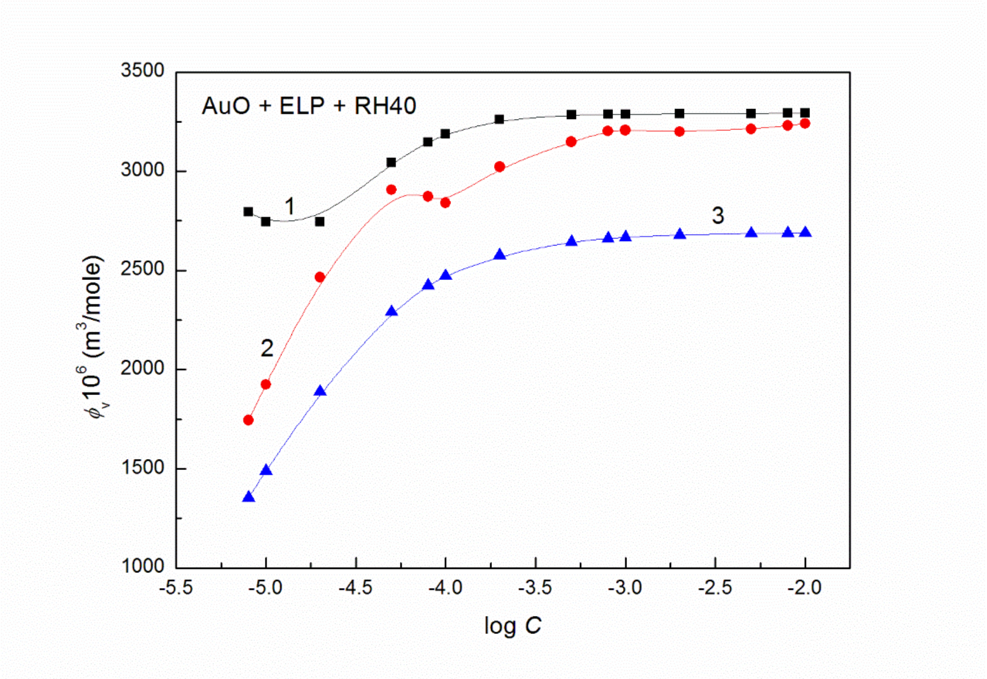

Figure S15. A plot of the apparent molar volume (ϕ_V) of aqueous solutions of ELP + RH40 (curve 1), AuO + ELP + RH40 mixture calculated from Eq. (15) (curve 2) and AuO + ELP + RH40 mixture calculated from Eq. (16) (curves 3) vs. the logarithm of ELP + RH40 concentration (log *C*).

Calculations using Equations (20) and (21).

The values of K_b and r calculated from Eq. (20) using the matched values of $F_f(\lambda)$,$F_b(\lambda)$, K_b and the established n (n = 1) and CMC [11] are presented in Table S1. The matched values of $F_f(\lambda)$, $F_b(\lambda)$ are given in Fig S16. As follows from Fig. S16 there is the maximum fluorescence intensity for auramine in the ELP micelles at λ = 500 nm. The emission maximum for the aqueous solution is difficult to determine from the available data due to the very weak signal intensity for the aqueous solution and the fluorescence

spectrum overlapping the Raman peak around 510 - 515 nm. In order to eliminate the influence of the Raman peak on the data analysis, the solvent spectrum (without auramine) should be measured with the identical instrument settings and the background spectrum thus measured should be subtracted from each of the analyzed spectra. Nevertheless, given that the fluorescence intensity of auramine is a measure of the microviscosity of an environment [4,5], it can be concluded that the relative (relative to water) microviscosity of the auramine solubilization site in the ELP micelles decreases with temperature. In other words, the microviscosity of the auramine solubilization site in the ELP micelles decreases with temperature faster than the microviscosity of water. Similarly, it can be seen that the value of the $\frac{K_b}{n}$ ratio decreases largely with the increasing temperature. Unfortunately, there is no information about the value of n and its temperature dependence. Most likely, the value of the constant K_b depends much more on the temperature than the value of n, and the observed large decrease in the value of the ratio $\frac{K_b}{n}$ with the temperature is mainly due to the decreasing role of hydrophobic interactions (entropy factor) with the increasing temperature. Nevertheless, due to the lack of information about the relationship $n = f\ (T)$, it is not possible to perform a thermodynamic analysis.

Based on the results of fitting the theoretical equations to the experimental data, it can be concluded that this fit is very good: the value of the R^2 coefficient is always greater than 0.998 (Table S1). A similar conclusion can be drawn based on the visual analysis of the fitting of the theoretical curves to the experimental data (curves for T = 293 K Fig. S17). It should be noted, however, that it was not possible to determine the value of the parameter r of the APN model for the tested system, i.e. the value corresponding to the best fit is also the upper limit for this parameter. Therefore, further calculations were performed without imposing the condition $r \leq 1$.

Table S1. The K_b values for ELP at the different temperatures; matched values of $F_f(\lambda)$,$F_b(\lambda)$, K_b and the established n (n = 1) and CMC [11].

T [K]	"n" [-]	K_b [dm³/mole]	CMC [mmole/dm³]	r [-]	R^2 [-]
293	1 (± 0)	0.4459 ± 0.0032	0.0214 (± 0.0000)	1.00 ± 0.55	0.998682
298	1 (± 0)	0.3795 ± 0.0022	0.0209 (± 0.0000)	1.00 ± 0.48	0.999224
303	1 (± 0)	0.3768 ± 0.0019	0.0203 (± 0.0000)	1.00 ± 0.43	0.999426
308	1 (± 0)	0.3408 ± 0.0024	0.0197 (± 0.0000)	1.00 ± 0.62	0.998996
313	1 (± 0)	0.3189 ± 0.0019	0.0191 (± 0.0000)	1.00 ± 0.55	0.999316
318	1 (± 0)	0.3001 ± 0.0030	0.0185 (± 0.0000)	1.00 ± 0.97	0.998130

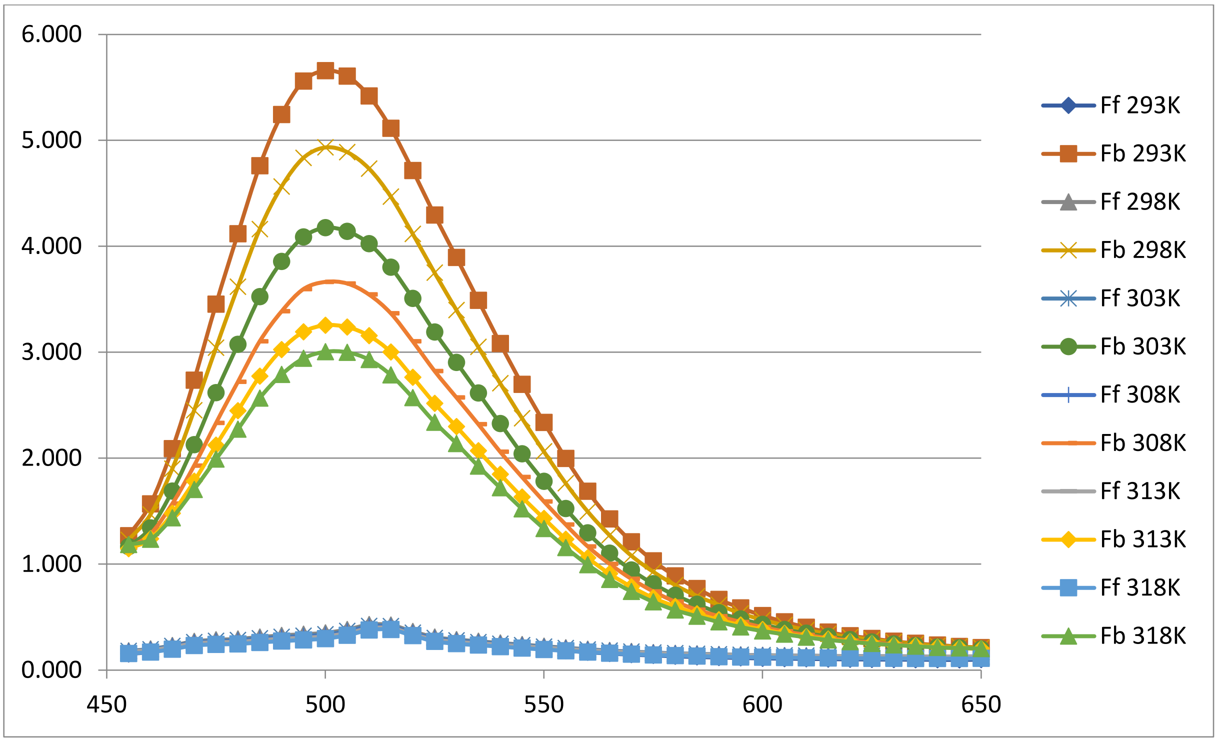

Figure S16. A matched values $F_f(\lambda)$, $F_b(\lambda)$ of fluorescence spectra of AuO in ELP solutions (*r* from 0 to 1).

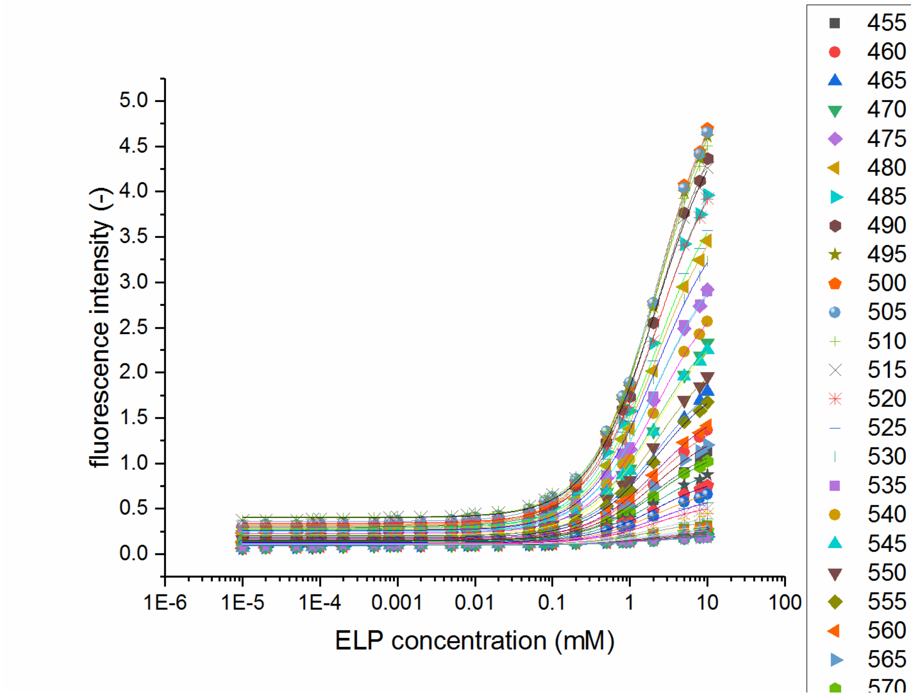

Figure S17. Fitting of the theoretical curves of the fluorescence intensity of AuO in ELP solutions to the experimental data (T = 293K, *r* from 0 to 1).

For this calculation the same matched and established magnitudes were used in Eq. (20) as mentioned above. The obtained values of $F_f(\lambda)$, $F_b(\lambda)$, K_b and r from Eq. (18) as well as the values of CMC taken from literature are presented in Table S2. Based on the calculations from Eq. (20) under the mentioned conditions it results that the values $F_f(\lambda)$, $F_b(\lambda)$), were very similar to those presented in Fig. S16. The obtained theoretical curves are more closer to the experimental data than in the first variant of the calculations (higher R^2 values). However, it is a purely mathematical effect because the r values obtained from the calculations have no physical sense. Similarly, the K_b values obtained in the second

variant of the calculations decreased slightly (compared to the results obtained in the first variant Table S1). However, the errors in their determination indicate that they do not differ significantly from 0 in most cases. Therefore, it should be stated that the change of the determined parameters depends on the imperfection of numerical methods to a greater extent than the physical meaning of the analyzed phenomenon. Thus, there is no actual improvement in the calculation results. Since the error in fitting the curves to the experimental data may result partly from the difference between the assumed and the actual values of CMC, another calculation variant was applied, in which CMC was also a numerically adjusted parameter. The calculated values of K_b, CMC and r from Eq. (20) at the matched $F_f(\lambda)$, $F_b(\lambda)$, K_b, r and CMC values for $n = 1$ are shown in Table S3. The spectra $F_f(\lambda)$ and $F_b(\lambda)$ obtained from Eq. (20) under these conditions are similar to those obtained in the first variant of the calculations. In turn, the K_b values were obtained with better precision in this variant of the calculation (Table S3) than in the second one (Table S2) and were similar to those in the first variant. (Table S1). Nevertheless, the obtained values and the precision of the r parameter calculations indicate that while the obtained values ensure the minimization of the objective function for the numerical procedure, they do not make much physical sense. The similar conclusion can be drawn for the CMC values. Among the CMC values calculated in this variant only that at T equal to 293 K is significantly different from 0 but at other temperatures the CMC values are at least of one order of magnitude smaller than those in the literature [11].

Table S2. The K_b values for ELP at the different temperatures; matched values of $F_f(\lambda)$, $F_b(\lambda)$, K_b, r and the established n ($n = 1$) and CMC [11].

T [K]	"n" [-]	K_b [dm³/mole]	CMC [mmole/dm³]	r [-]	R^2 [-]
293	1 (± 0)	0.4300 ± 5.2619	0.0214 (± 0.0000)	1 598 ± 2.38E+7	0.999159
298	1 (± 0)	0.3700 ± 1.9988	0.0209 (± 0.0000)	50 700 ± 1,11E+10	0.999494
303	1 (± 0)	0.3742 ± 0.0028	0.0203 (± 0.0000)	64 ± 35	0.999604
308	1 (± 0)	0.3330 ± 0.0020	0.0197 (± 0.0000)	6,94E+15	0.999201
313	1 (± 0)	0.3121 ± 3.6595	0.0191 (± 0.0000)	1 869 ± 3.07E+07	0.999501
318	1 (± 0)	0.2941 ± 3.1185	0.0185 (± 0.0000)	11 300 ± 1.07E+09	0.998418

Table S3. The K_b values for ELP at the different temperatures; matched values of $F_f(\lambda)$, $F_b(\lambda)$, K_b, r, CMC and the established n ($n = 1$).

T [K]	"n" [-]	K_b [dm³/mole]	CMC [mmole/dm³]	r [-]	R^2 [-]
293	1 (± 0)	0.4463 ± 0.0026	0.00003 ± 0.00323	0.0747 ± 1.19E+09	0.999118
298	1 (± 0)	0.3766 ± 0.0018	0.00133 ± 0.00139	0.0436 ± 5.41E+07	0.999469
303	1 (± 0)	0.3744 ± 0.0017	0.00472 ± 0.00126	0.0014 ± 0	0.999563
308	1 (± 0)	0.3384 ± 0.0021	0.00083 ± 0.00189	0.0058 ± 5.56E+07	0.999185
313	1 (± 0)	0.3169 ± 0.0016	0.00052 ± 0.00161	0.0068 ± 4.53E+08	0.999489
318	1 (± 0)	0.2981 ± 0.0027	0.00074 ± 0.00285	0.0094 ± 0	0,998392

It should be noted that taking into account the results of the calculations using Eq. (20) under the mentioned conditions in the first, second and third variants, respectively it is impossible to obtain meaningful values of the APN model parameters [30,31] for the studied surfactant-solubilizate system based on the available experimental data. This is due to both the inevitable experimental errors, the specificity of the tested system and the specificity of the APN model itself. This is evident, for example, from the dependence between the visual variability of the fluorescence intensity and the surfactant concentration (Fig. S18). Based on this dependence it can be stated that taking into account the typical experimental errors, the beginning of the changes of the fluorescence intensity took place at the beginning of the co-ordinate system. This indicate that taking into account the experimental error limit CMC = 0. Even more so, it is not possible to determine a meaningful value for the parameter r because its value depends only on the nature of changes in the observed signal in the concentration range very close to the CMC value. One of the possible ways to deal with the above situation is to use the APN model [28,29] while giving up its advantages, i.e. assuming the absence of a transition region around the

CMC - that is, by carrying out calculations assuming the value of r = 0. Then the APN model behaves as in the simplest, borderline approach to micellization - i.e. below CMC all surfactant is in monomeric form, and each portion of the surfactant above the CMC value is in in micellar form. In the other words:

- for $C_{S,0} \leq cmc$: $C_{S,M} = 0$; $C_{S,1} = C_{S,0}$;

- for $C_{S,0} \geq cmc$: $C_{S,M} = C_{S,0} - cmc$; $C_{S,1} = cmc$;

Due to the mathematical construction of the APN model and the specificity of numerical methods, calculations for r = 0 cannot be performed, but correct results can be obtained assuming very small value of this parameter, e.g. $r = 1 \times 10^{-6}$. Thus taking into account this value of r the fourth variant of the calculations using Eq. (20) was done under the conditions that $F_f(\lambda)$, $F_b(\lambda)$ and K_b are matched and established n = 1 and $r = 1 \times 10^{-6}$ as well as CMC values were taken from the literature [11].

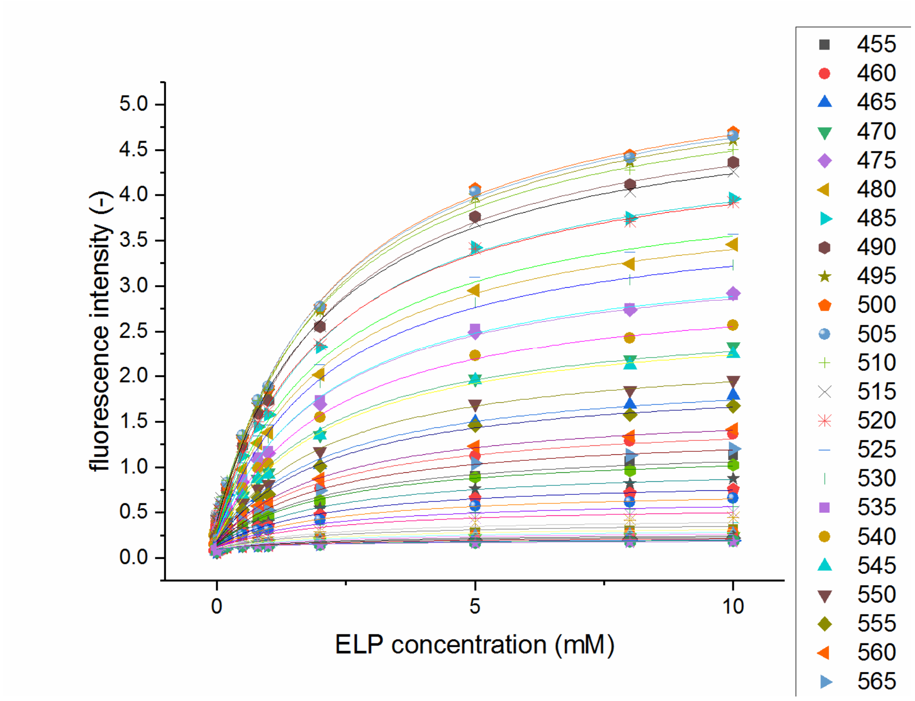

Figure S18. A plot of the visual variability the fluorescence intensity of AuO in ELP solutions vs. ELP concentration (T = 293K).

The values obtained from the calculations are presented in Table S4 and Figs. S19 and S20. Based on these values the same conclusions as presented above can be drawn. To calculate $F_W(\lambda)$, $F_M(\lambda)$, K_{MW}, r values from Eq. (21) also different variants of the matched and established values were used.

In the first variant the calculations were made assuming that $F_W(\lambda)$, $F_M(\lambda)$ K_{MW} and r are matched and $V_{S,M}^{m}$ and CMC established and taken from the literature [10,11] (Table S5). The second variant was realized at the same assumption as the first variant (Table S6). In turn the third variant of the calculations of $F_W(\lambda)$, $F_M(\lambda)$, K_{MW}, r and CMC from Eq. (21) was made at the assumption that $F_W(\lambda)$, $F_M(\lambda)$, K_{MW}, r and CMC are matched but $V_{S,M}^{m}$ established (Table S7). The fourth variant of the calculations was done using Eq. (21) at the assumption that $F_W(\lambda)$, $F_M(\lambda)$ and K_{MW} are matched and $V_{S,M}^{m}$ and CMC are established and taken from the literature [11], and $r = 1 \times 10^{-6}$ (Table S8 and Fig. S21). The results obtained from the calculations from Eq. (21) allow to draw the same conclusion as those presented above.

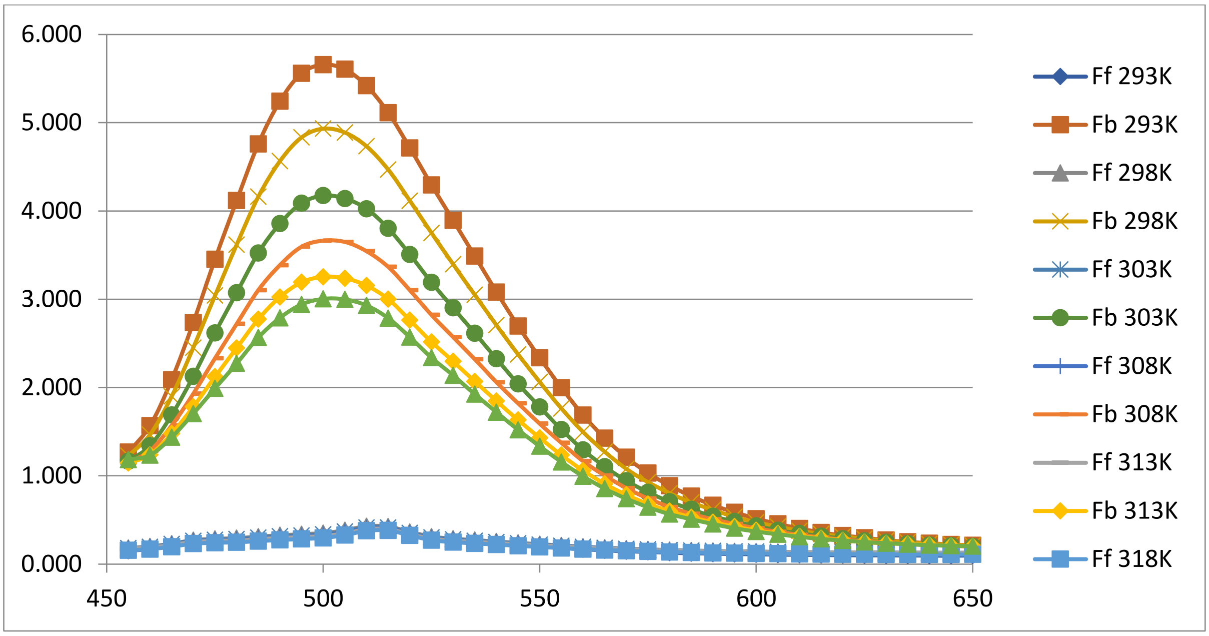

Figure S19. A matched values $F_f(\lambda)$, $F_b(\lambda)$ of fluorescence spectra of AuO in ELP solutions (r = 10^{-6}).

Table S4. The K_b values for ELP at the different temperatures; matched values of $F_f(\lambda)$, $F_b(\lambda)$, K_b and the established n = 1, r = 10^{-6}, CMC.

T [K]	"n" [-]	K_b [dm³/mole]	CMC [mmole/dm³]	r [-]	R^2 [-]
293	1 (± 0)	0.4395 ± 0.0031	0.0214 (± 0.0000)	10^{-6} (± 0)	0.998688
298	1 (± 0)	0.3785 ± 0.0022	0.0209 (± 0.0000)	10^{-6} (± 0)	0.999208
303	1 (± 0)	0.3760 ± 0.0019	0.0203 (± 0.0000)	10^{-6} (± 0)	0.999411
308	1 (± 0)	0.3401 ± 0.0023	0.0197 (± 0.0000)	10^{-6} (± 0)	0.998984
313	1 (± 0)	0.3182 ± 0.0018	0.0191 (± 0.0000)	10^{-6} (± 0)	0.999307
318	1 (± 0)	0.2993 ± 0.0029	0.0185 (± 0.0000)	10^{-6} (± 0)	0.998109

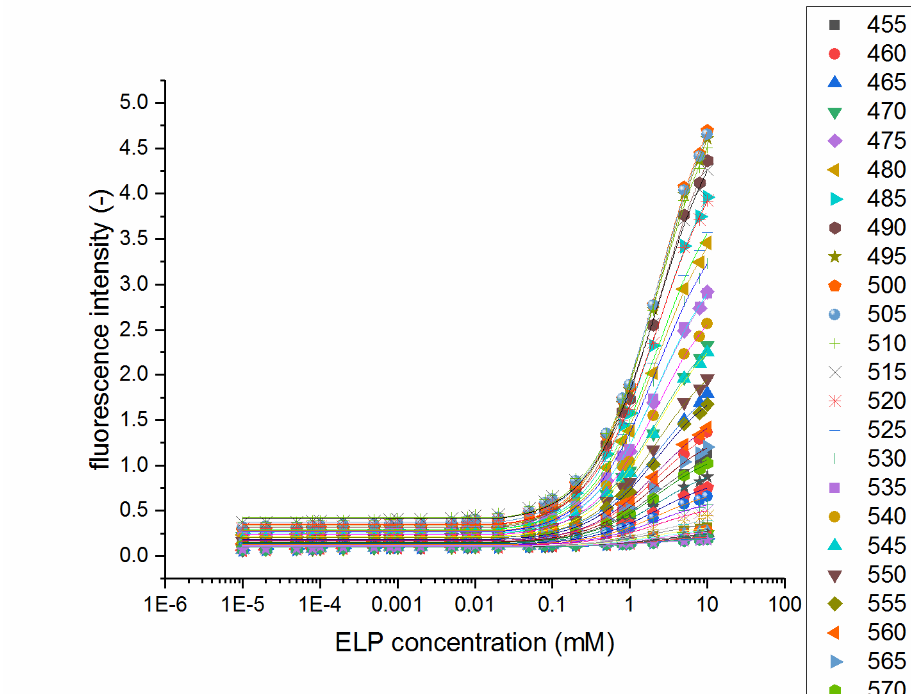

Figure S20. Fitting of the theoretical curves of the fluorescence intensity of AuO in ELP solutions to the experimental data (T = 293K, r = 10^{-6}).

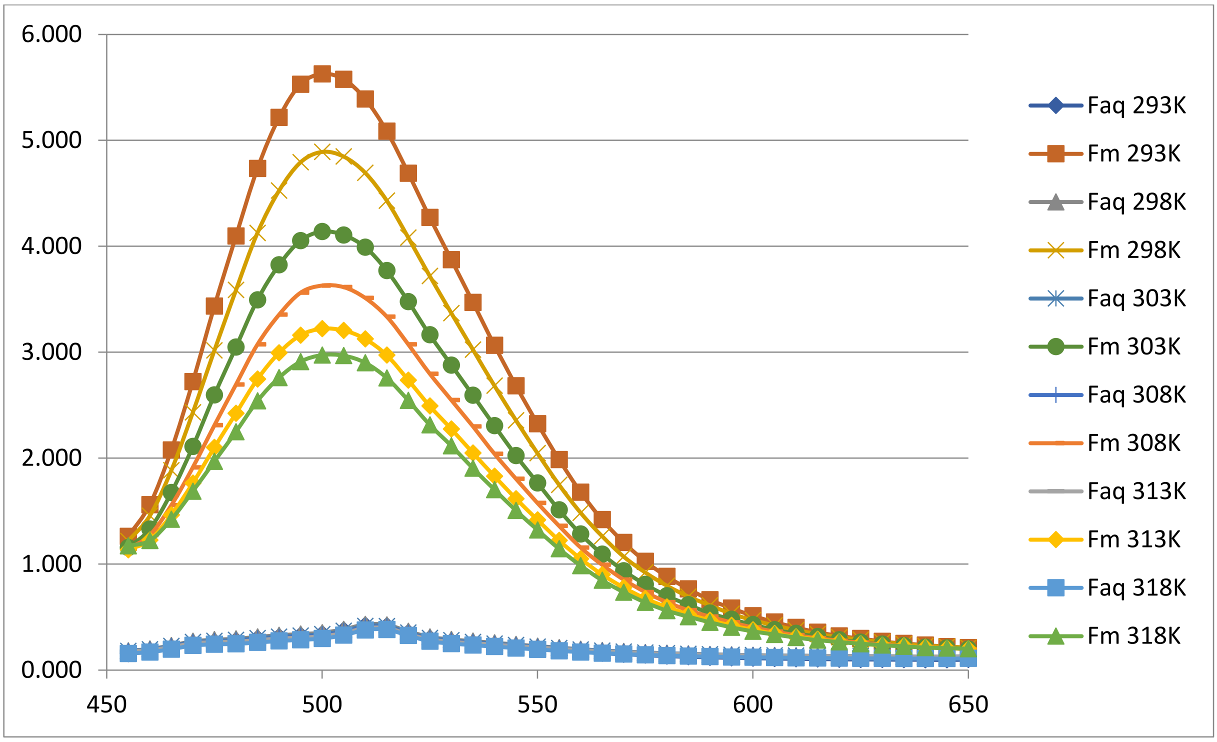

Figure S21. A matched values $F_f(\lambda)$, $F_b(\lambda)$ of fluorescence spectra of AuO in ELP solutions (established parameters: $V_{S,M}^{m}$, CMC, $r = 10^{-6}$).

Table S5. The K_{MW} values for ELP at the different temperatures; matched values of $F_W(\lambda)$, $F_M(\lambda)$, K_{MW}, r and the established $V_{S,M}^{m}$, CMC.

T [K]	$V_{S,M}^{m}$ [cm³/mole]	K_{MW} [-]	CMC [mmole/dm³]	r [-]	R^2 [-]
293	3 706.54 (± 0.00)	121.1 ± 0.9	0.0214 (± 0.0000)	1.00 ± 0.54	0.998693
298	3 710.84 (± 0.00)	103.4 ± 0.6	0.0209 (± 0.0000)	1.00 ± 0.48	0.999223
303	3 716.04 (± 0.00)	102.4 ± 0.5	0.0203 (± 0.0000)	1.00 ± 0.43	0.999426
308	3 722,06 (± 0.00)	92.6 ± 0.6	0.0197 (± 0.0000)	1.00 ± 0.62	0.998996
313	3 728.87 (± 0.00)	86.2 ± 0.5	0.0191 (± 0.0000)	1.00 ± 0.55	0.999316
318	3 736.39 (± 0.00)	81.3 ± 0.8	0.0185 (± 0.0000)	1.00 ± 0.97	0.998130

Table S6. The K_{MW} values for ELP at the different temperatures; matched values of $F_W(\lambda)$, $F_M(\lambda)$, K_{MW}, r (only lower limit) and the established $V_{S,M}^{m}$, CMC.

T [K]	$V_{S,M}^{m}$ [cm³/mole]	K_{MW} [-]	CMC [mmole/dm³]	r [-]	R^2 [-]
293	3 706.54 (± 0.00)	117 ± 805	0.0214 (± 0.0000)	4 047 ± 8.92E+07	0.999159
298	3 710.84 (± 0.00)	101 ± 533	0.0209 (± 0.0000)	27 326 ± 3.19E+09	0.999494
303	3 716.04 (± 0.00)	101.7 ± 0.7	0.0203 (± 0.0000)	64 ± 35	0.999604
308	3 722,06 (± 0.00)	90.5 ± 0.0	0.0197 (± 0.0000)	4.78E+18 ± 0.0138	0.999201
313	3 728.87 (± 0.00)	84.7 ± 661	0.0191 (± 0.0000)	1 673 ± 1.64E+07	0.999501
318	3 736.39 (± 0.00)	81.4 ± 770.1	0.0185 (± 0.0000)	2.66E+09 ± 5.44E+19	0.998368

Table S7. The K_{MW} values for ELP at the different temperatures; matched values of $F_W(\lambda)$, $F_M(\lambda)$, K_{MW}, r, CMC and the established $V_{S,M}^{m}$.

T [K]	$V_{S,M}^{m}$ [cm³/mole]	K_{MW} [-]	CMC [mmole/dm³]	r [-]	R^2 [-]
293	3 706.54 (± 0.00)	125.7 ± 0.8	0.00132 (± 0.00167)	0.0226 ± 0.0000	0.998934
298	3 710.84 (± 0.00)	105.5 ± 0.5	0.00406 (± 0.00142)	0.0212 ± 4.66E+06	0.999406
303	3 716.04 (± 0.00)	101.5 ± 0.4	0.00178 (± 0.00121)	0.0111 ± 2.89E+06	0.999583
308	3 722,06 (± 0.00)	91.9 ± 0.6	0.00098 (± 0.00188)	0.0049 ± 6.98E+06	0.999184
313	3 728.87 (± 0.00)	86.0 ± 0.4	0.00160 (± 0.00153)	0.0310 ± 1.07E+06	0.999481
318	3 736.39 (± 0.00)	80.9 ± 0.7	0.00003 (± 0.00433)	0.0351 ± 0.0000	0.998400

Table S8. The K_{MW} values for ELP at the different temperatures; matched values of $F_W(\lambda)$, $F_M(\lambda)$, K_{MW} and the established $V_{S,M}^m$, CMC, r = 10^{-6}.

T [K]	$V_{S,M}^m$ [cm³/mole]	K_{MW} [-]	CMC [mmole/dm³]	r [-]	R^2 [-]
293	3 706.54 (± 0.00)	119.6 ± 0.8	0.0214 (± 0.0000)	10^{-6} (± 0)	0.998688
298	3 710.84 (± 0.00)	103.0 ± 0.6	0.0209 (± 0.0000)	10^{-6} (± 0)	0.999208
303	3 716.04 (± 0.00)	102.2 ± 0.5	0.0203 (± 0.0000)	10^{-6} (± 0)	0.999411
308	3 722,06 (± 0.00)	92.4 ± 0.6	0.0197 (± 0.0000)	10^{-6} (± 0)	0.998984
313	3 728.87 (± 0.00)	86.3 ± 0.5	0.0191 (± 0.0000)	10^{-6} (± 0)	0.999307
318	3 736.39 (± 0.00)	81.1 ± 0.8	0.0185 (± 0.0000)	10^{-6} (± 0)	0.998109
